# Enhancement of the thermoelectric figure of merit in the Dirac semimetal Cd_3_As_2_ by band-structure and -filling control

**DOI:** 10.1080/14686996.2024.2412971

**Published:** 2024-10-08

**Authors:** Markus Kriener, Takashi Koretsune, Ryotaro Arita, Yoshinori Tokura, Yasujiro Taguchi

**Affiliations:** a RIKEN Center for Emergent Matter Science (CEMS), Wako, Japan; b Department of Physics, Tohoku University, Miyagi, Japan; c Research Center for Advanced Science and Technology, University of Tokyo, Tokyo, Japan; d Department of Applied Physics and Quantum-Phase Electronics Center (QPEC), University of Tokyo, Tokyo, Japan; e Tokyo College, University of Tokyo, Tokyo, Japan

**Keywords:** Thermoelectric materials, figure of merit, band structure engineering, band filling, Dirac semimetal, Cd_3_As_2_

## Abstract

Topological materials attract a considerable research interest because of their characteristic band structure giving rise to various new phenomena in quantum physics. Besides this, they are tempting from a functional materials point of view: Topological materials bear potential for an enhanced thermoelectric efficiency because they possess the required ingredients, such as intermediate carrier concentrations, large mobilities, heavy elements etc. Against this background, this work reports an enhanced thermoelectric performance of the topological Dirac semimetal Cd_3_As_2_ upon alloying the trivial semiconductor Zn_3_As_2_. This allows to gain fine-tuned control over both the band filling and the band topology in Cd_3-*x*_Zn_*x*_As_2_. As a result, the thermoelectric figure of merit exceeds 0.5 around x=0.6 and x=1.2 at elevated temperatures. The former is due to an enhancement of the power factor, while the latter is a consequence of a strong suppression of the thermal conductivity. In addition, in terms of first-principle band structure calculations, the thermopower in this system is theoretically evaluated, which suggests that the topological aspects of the band structure change when traversing x=1.2.

## IMPACT STATEMENT

Controlling the band-filling of a topological Dirac semimetal with a high mobility is demonstrated to be a promising strategy to achieve a large thermoelectric figure of merit *ZT*.

## GRAPHICAL ABSTRACT

**Figure:**
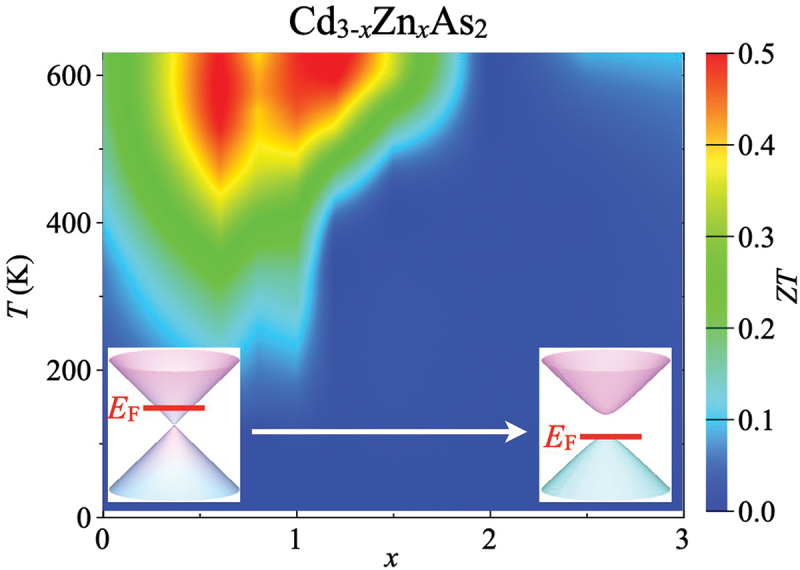

## 1. Introduction

The development and exploration of sustainable energy devices is a timely task in materials design and research. One important issue is that a substantial amount of input energy is transferred into heat and lost irretrievably in many applications. To overcome this, the Seebeck effect offers a solution: It allows to regain electrical energy by conversion from waste heat.

To quantify the conversion efficiency of bulk materials, the dimensionless figure of merit is used: $\mathrm{ZT}=S^{2}T/(\rho \kappa )$ with the thermopower S, the resistivity ρ, and the thermal conductivity κ (T denotes the temperature). The goal is to maximize ZT [1–6]. However, the involved quantities ρ, S, and κ are strongly interrelated and difficult to tune independently, which explains why to date only a few materials with some what large ZT values were found. Hence, there is a continuing demand on identifying new promising materials which ideally exhibit large ZT values over a wide temperature range.

Various strategies and concepts to enhance ZT have been proposed and employed, such as exploiting strong correlation effects [7,8], carrier concentration control [9–11], band structure engineering (band convergence and resonance level formation) [4,5,11–20], magnetic interactions [21,22], utilizing physical pressure [23], or nanostructured devices [13,24,25]. ‘Phonon glass + electron crystal’ is often referred to as the guiding principle, i.e., a material should possess ideally independent charge- and heat-transport channels to obtain small ρ and κ values simultaneously [3,26].

Against this background, it is interesting that some materials with promising thermoelectric features have attracted attention from a different point of view in recent years: they were identified to possess a topologically nontrivial band structure, such as the prototypical thermoelectric Bi_2_Te_3_ [27–29]. The coincidence of topological nontriviality and thermoelectric efficiency is, however, not surprising since both phenomena partly share the same ingredients: intermediate carrier concentrations, large mobilities, heavy elements, or narrow band gaps [30,31]. This motivated us to focus on the Dirac semimetal Cd_3_As_2_ [32–39]. Besides its topological features, it has long been known for its large room-temperature mobility of $∼10^{4}-10^{7}$ cm^2^V^−1^s^−1^ while the electron charge carrier concentration lies at moderate $∼10^{18}$cm^−3^ [34,40]. Moreover, its thermal conductivity was reported to be as small as 2.5–4 WK^−1^m^−1^ at 300 K with an extremely tiny lattice contribution $\kappa_{\mathrm{ph}}∼1$ WK^−1^m^−1^ [41–45]. Consistently, a room-temperature figure of merit ZT∼0.1 was reported [43,46], with an additional slight enhancement upon shallow Cr doping [43]. In thin films of Cd_3_As_2_, enhanced values of the thermopower and the power factor $S^{2}/\rho$ were reported at low temperatures as a function of the film thickness [47]. There is also theoretical support that topological materials such as Cd_3_As_2_ are promising playgrounds to look for a reasonable thermoelectric performance [48–50].

Here, we chose to mix the 4d system Cd_3_As_2_ with its lighter 3d counterpart Zn_3_As_2_ because partial Zn doping modifies both the band filling and the band structure: A hole carrier concentration of $∼10^{17}cm^{-3}$ and a much smaller mobility of a few 10 cm^2^V^−1^s^−1^ reported for Zn_3_As_2_ suggest that alloying offers to fine tune and gain control of these quantities and, hence, the figure of merit of Cd_3-*x*_Zn_*x*_As_2_ across the charge neutrality point [51–54]. In addition, the question at what x the topological band inversion in Cd_3_As_2_ switches back to normal band order is tempting since Zn_3_As_2_ is theoretically a trivial band insulator. Recent reports allocate this change around x∼1.1 for the bulk system [54] while it was seen at a smaller x∼0.6 in thin-film studies [55–57].

In a preceding work [58], where we only focussed on the thermoelectric performance of Cd_3-*x*_Zn_*x*_As_2_ below room temperature in the very limited Zn concentration range 0≤x≤1.2, we reported that ZT reaches ∼0.3±0.1 for x∼0.8−1.0. This and the sizable slope of ZT(T) at room temperature motivated the present work, in which we largely extend our previous study to approximately 650 K and target the whole solid solution 0≤x≤3. Moreover, in the present work we also modeled the band structure on the basis of first-principle calculations for Cd_3_As_2_ and Zn_3_As_2_, and subsequently calculated the temperature dependence of the thermopower for various carrier concentrations for comparison with the experimental results to give an insight into the question where the topological aspects of the band structure may change.

The examination of this much wider phase space in terms of temperature and Zn concentration allowed to identify two different x ranges where ZT becomes large: At 600 K, ZT exceeds 0.5 around x∼0.6 and ∼1.2. In addition, the comparison of theoretical and experimental thermopower data yielded strong support for the conclusion that the topological band order vanishes between x=1.0 and 1.2.

## 2. Results

**Figure 1.**
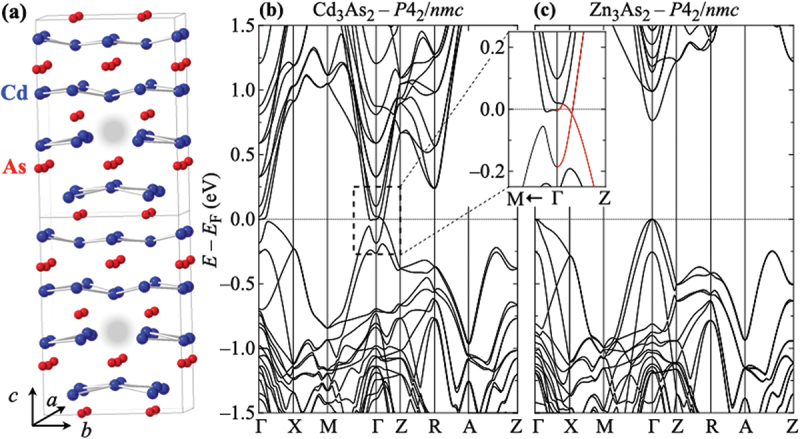
(a) Schematic of Cd_3_As_2_ in its tetragonal $P4_{2}/\mathrm{nmc}$ structure (‘β-Cd_3_As_2_’). in this setting, the characteristic voids (gray shadows) on the Cd sites arrange along the a or b axis, cf. Ref. [33]. Band structures of (b) Cd_3_As_2_ and (c) Zn_3_As_2_ based on the structural β modification. In the inset, the band structure of Cd_3_As_2_ around the Γ point is magnified with the Dirac-like band dispersion highlighted in red.

We start with a brief discussion of the structure-related situation in Cd_3-*x*_Zn_*x*_As_2_. At ambient conditions, a series of structural modifications is realized as a function of x. Moreover, for the end compounds Cd_3_As_2_ and Zn_3_As_2_, two different modifications are discussed in the literature, referred to as α phase in this work: the body-centered tetragonal space groups $I4_{1}/\mathrm{acd}$ (no. 142) [33,51] and $I4_{1}/\mathrm{cd}$ (no. 110) [59,60]. According to a recent publication, the former one seems realized in Cd_3_As_2_ [33]. In the Zn concentration range 0.6≤x≤1.2, the system crystallizes in the simple tetragonal space group $P4_{2}/\mathrm{nmc}$ (no. 137, β phase) and for 1.5≤x≤2.5 it takes the closely related superstructure $I4_{1}/\mathrm{amd}$ (no. 141; $\beta^{\prime }$ phase). This complex situation is discussed in more detail in Section S1 in the accompanying Supplemental Material. A schematic plot of the β-type crystal structure is shown in Figure 1(a).

Figures 1(b,c) summarize the results of band-structure calculations for the two pristine materials Cd_3_As_2_ and Zn_3_As_2_, respectively. These are based on the β phase which was chosen for an easy comparison with experimental data for alloyed samples. For Cd_3_As_2_ and Zn_3_As_2_, the main features of the β phase are the same as in the α modifications. Notably, Cd_3_As_2_ remains a semimetal and exhibits a band crossing with Dirac-like dispersion near the Γ point close to the Fermi energy as can be seen in the magnified inset in Figure 1. The opposite end compound Zn_3_As_2_ is a trivial band insulator in both structural modifications, while Zn_3_As_2_ is conductive due to unintentionally doped holes in reality.

**Figure 2.**
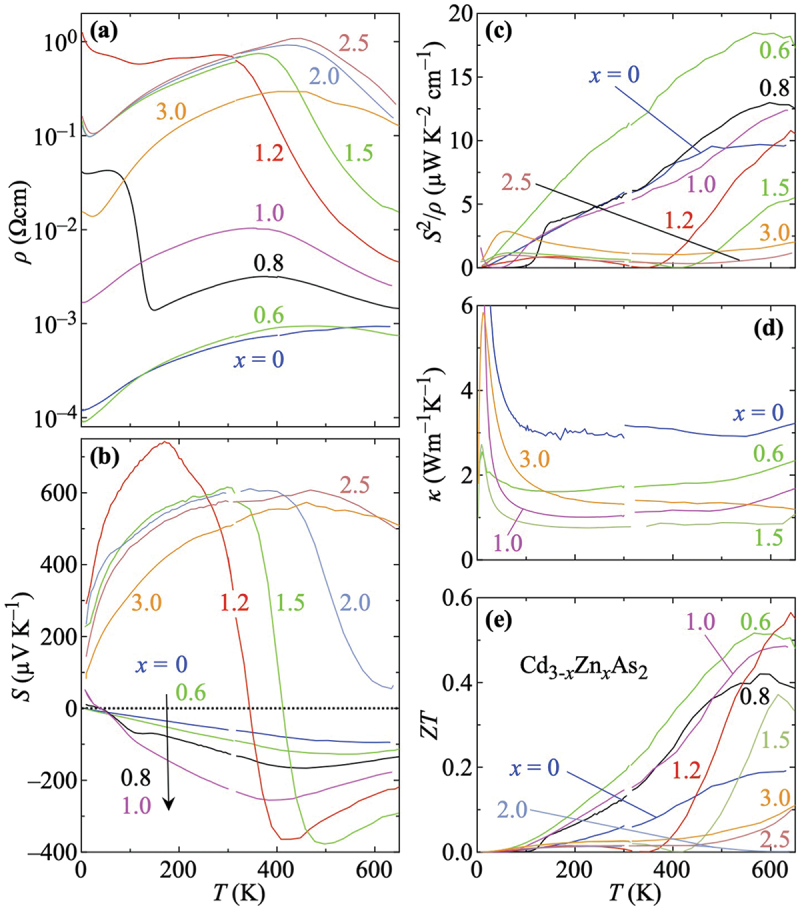
Temperature dependence of the thermoelectric properties of the solid solution Cd_3-*x*_Zn_*x*_As_2_ (0≤x≤3): (a) resistivity ρ, (b) thermopower S, (c) power factor $S^{2}/\rho$, (d) thermal conductivity κ, and (e) figure of merit $\mathrm{ZT}=S^{2}T/(\rho \kappa )$. In (c) the data for x=2.0 (overall very small $S^{2}/\rho$) and in (d) the data for some x are omitted for clarity. The latter can be found in section S3 in the Supplemental Material.

Figure 2 summarizes the temperature dependence of all quantities related to the figure of merit of Cd_3-*x*_Zn_*x*_As_2_. The resistivity ρ(T) is shown in Figure 2(a). Except for the strong increase for T<∼150 K in the data for x=0.8, all samples with 0.6≤x≤1.0 exhibit a metallic-like temperature dependence up to a temperature $T_{\max}$ and then decrease. As a function of x, $T_{\max}$ decreases monotonically, suggesting that there is also a maximum outside the targeted temperature range in ρ for x=0, which tends to saturate above 600 K. As for the low-temperature increase in the data of x=0.8, we occasionally observe such metal-insulator transitions below room temperature for x≤1.2, cf. Ref. [58] and Section S7 in the Supplemental Material.

For x≥1.2, the resistivity of Cd_3-*x*_Zn_*x*_As_2_ is strongly enhanced and exhibits maxima, which shift up to x=2.5 toward higher temperatures. The data for x≥1.5 look qualitatively similar while the absolute values of the resistivity increases with x except for Zn_3_As_2_, in rough agreement with the literature [51]. The maxima imply that additional charge carriers are activated at elevated temperatures, possibly leading to bipolar conduction. In Section S5 in the Supplemental Material, we present an Arrhenius analysis of the high-temperature resistivity.

Figure 2(b) summarizes the corresponding thermopower S(T) data. For all samples x≤1, we observe a negative S except at very low temperatures. There are also distinct extrema whose temperatures $T_{1}$ decrease monotonically with x. As expected for these metallic samples, S exhibits roughly a linear temperature dependence below $T_{1}$ with some deviations for x=0.8 and 1.0 at low temperatures. Above $T_{1}$, the absolute value of S decreases. Upon further increasing x, there is a clear qualitative and quantitative change. For x=1.2 and 1.5, S exhibits two extrema at $T_{1}$ and $T_{2}\;(<T_{1})$. In between the sign of S changes from positive to negative. For $T>T_{1}$, S of these two samples is qualitatively similar to what is observed for x≤1. For x=2.0, there is no sign change of S any more but the two extrema as characteristic features are still visible although $T_{1}(x)$ has almost shifted out of the examined temperature range. The latter seems to be the case for x=2.5 and 3, each of which exhibits only a broad extremum up to 650 K. The x dependencies of these characteristic temperatures in ρ(T) and S(T) are replotted in Figure 5.

Corresponding power-factor data $S^{2}/\rho (T)$ are plotted in Figure 2(c), which is strongly enhanced over the entire temperature range studied when going from x=0 to 0.6. For x=0.8 and 1.0, $S^{2}/\rho$ is similar to x=0 up to above room temperature and exceeds it toward 650 K. For x=1.2 and 1.5, $S^{2}/\rho$ are much smaller than for x≤1 up to above room temperature, reflecting the large resistivities of these samples. Toward 650 K, a steep increase is observed for both and, in the case of x=1.2, it even exceeds x=0. For the sample with x=2.0, $S^{2}/\rho$ is rather small throughout the examined temperature range [not shown for clarity in Figure 2(c)]. For x=2.5 and 3.0, there is a slight increase above ∼400K.

Figure 2(d) summarizes thermal conductivity κ(T) data. Besides the increases below ∼100K, κ of Cd_3-*x*_Zn_*x*_As_2_ is remarkably small. For x=0, we observe κ<4 WK^−1^m^−1^. Moreover, κ is further suppressed when introducing Zn and takes its minimum for x=1.5 in most of the temperature range. Toward Zn_3_As_2_, κ increases again but remains below that for x=0.

The figure of merit ZT(T) for all examined samples is shown in Figure 2(e). For 0≤x≤1.0 we find relatively large ZT values from well below room temperature, which further increase as a function of temperature up to about 500 K. Above, ZT exhibits a maximum or an onset of a maximum. For x=1.2 and 1.5, ZT remains small up to ∼350 K and 420 K, respectively. Upon further increasing temperature, these two samples also exhibit comparably large ZT values. Interestingly, ZT of x=1.2 eventually exceeds the data of x=0.6, although only above 600 K. For x=2.5 and 3.0, we find slightly enhanced ZT values above ∼500 K.

## 3. Discussion

### 3.1. The figure of merit

**Figure 3.**
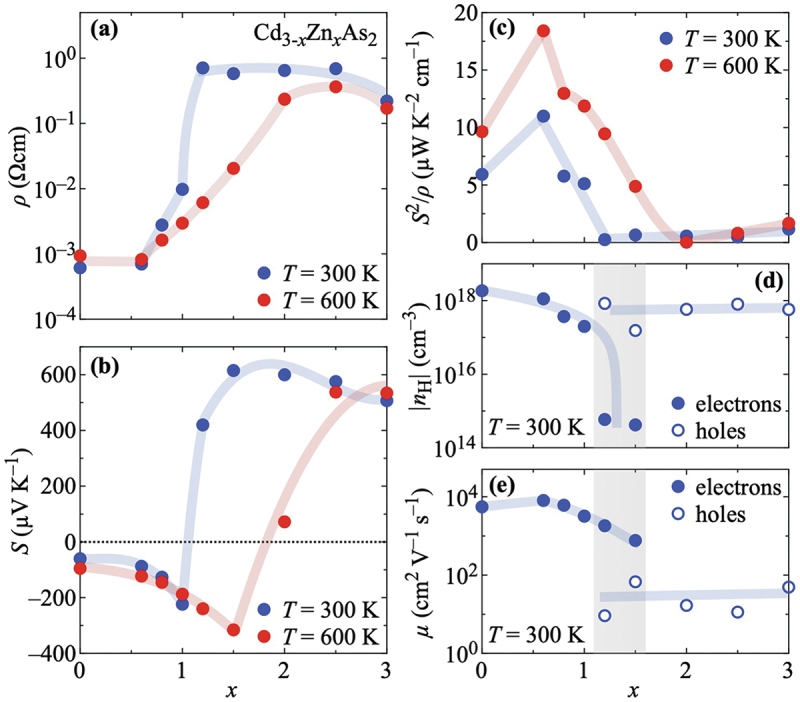
Zn concentration x dependence of (a) the resistivity ρ, (b) the thermopower S, (c) the power factor $S^{2}/\rho$, (d) the charge carrier concentration $|n_{{\;}_{H}}|$ as estimated from field-dependent Hall-resistivity data, and (e) the corresponding charge carrier mobility μ of Cd_3-*x*_Zn_*x*_As_2_ (0≤x≤3). Blue (red) symbols indicate data taken at 300 K (600 K). Blueish and reddish bold lines are guides to the eyes, and the gray shaded areas in (d) and (e) indicate the Zn concentration range in which electrons (filled symbols) and holes (open symbols) apparently coexist as charge carriers, see text.

**Figure 4.**
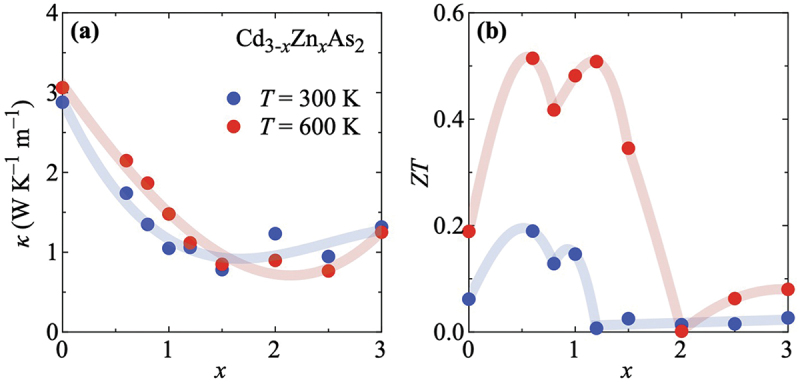
Zn concentration x dependence of (a) the thermal conductivity and (b) the dimensionless figure of merit ZT of Cd_3-*x*_Zn_*x*_As_2_ (0≤x≤3). Blue (red) symbols denote data taken at 300 K (600 K). Blueish and reddish bold lines are guides to the eyes.

Figures 3 and 4 summarize several important quantities at 300 K and 600 K as a function of the Zn concentration x. In Figure 3(a), the strong enhancement of the resistivity ρ(x) with x is well recognizable in the 300-K data across x=1. In the 600-K data, this increase is less steep. A consequence of the maxima in ρ(T) is that, except for x≤0.6, the resistivity values at 600 K are smaller than those at 300 K.

Figure 3(b) contains the respective thermopower data S(x). The sign change of S from negative (x≤1.0) to positive (x≥2.0) is clearly discernible. The occurrence of extrema above 300 K in S(T) leads to comparable absolute values of the thermopower at 300 K and 600 K for x≤1.0 and x≥2.5. In between, the two extrema in S(T) cause very different values of S at 300 K and 600 K. For x=1.5, S changes from about +600 μVK${\;}^{-1}$ to −300 μVK${\;}^{-1}$.

These observations are reflected in the power-factor data $S^{2}/\rho (x)$ shown in Figure 3(c). The still relatively small resistivity and large absolute thermopower for x≤1.0 manifests itself as a strong enhancement of $S^{2}/\rho$ with a peak at x=0.6 at both temperatures shown. The recovery of the thermopower for x≥2.5 results only in a slight increase of $S^{2}/\rho$ toward Zn_3_As_2_ due to the large resistivity.

Figure 3(d,e) summarize the x dependencies of the absolute values of the charge carrier concentration $|n_{{\;}_{H}}|$ as estimated from magnetic field B-dependent Hall-resistivity measurements $\rho_{\mathrm{yx}}(B)$ at 300 K and the corresponding mobility $\mu =1/(|n_{{\;}_{H}}|\mathrm{\rho e})$ with the elementary charge e, respectively.

When introducing Zn into the system, the electronic transport remains initially dominated by electrons. Their concentration of $∼10^{18}$cm${\;}^{-3}$ for x=0 is reduced by approximately one order of magnitude for x=1.0, i.e. the band filling (with electrons as charge carriers) is reduced when introducing Zn into Cd_3_As_2_. Upon further alloying, the drop of the electron concentration is accelerated and falls below $10^{15}cm^{-3}$ for x=1.2 and 1.5. At the same time, holes with a concentration of $10^{17}$ – $10^{18}$cm^−3^ also start to contribute to the electronic transport, highlighting the change in the nature of the band filling as a function of x. The simultaneous presence of holes and electrons causes strongly non-linear Hall-resistivity data, cf. Figures S13 and S14 in Section S4 in the Supplemental Material. The temperature dependence of both carrier types for these two samples can be found therein in Figure S15. For x≥2.0, holes are the dominating charge carriers. Their concentration and, hence, the band filling does not change much. A simple phenomenological model of the band-filling evolution fitting to these observations is presented in Section S6 in the Supplemental Material. Another interesting point here is that the mobility of pristine Cd_3_As_2_ is apparently smaller than for x=0.6, above which it decreases again as a function of x. We will come back to this feature later.

Importantly, the electrons governing the transport on the Cd-rich side of the phase diagram maintain their large mobility which amounts to several 1000 cm${\;}^{2}$V${\;}^{-1}$s${\;}^{-1}$ for x≤1.2, cf. Figure 3(e). By contrast, the hole carriers for all samples x≥1.2 exhibit low mobility values of less than 70 cm${\;}^{2}$V${\;}^{-1}$s${\;}^{-1}$, i.e. Zn alloying removes high-mobility electrons and replaces them with low-mobility holes while the hole concentrations for x≥1.2 are on a similar order of magnitude as those of the electrons for x≤1.0.

As seen in Figure 4(a), Cd_3-*x*_Zn_*x*_As_2_ exhibits an overall small κ(x) at both 300 K and 600 K. The values at 300 K are smaller (larger) than those at 600 K for x≤1.5 (x≥2.0). The largest values of κ∼3 WK${\;}^{-1}$m${\;}^{-1}$ are found in pure Cd_3_As_2_ in agreement with previous reports in the literature [41–45]. Upon Zn alloying, κ is further suppressed. We observe the smallest thermal conductivity κ<1 WK${\;}^{-1}$m${\;}^{-1}$ at 300 K for x=1.5 and at 600 K for x=2.5. This additional suppression of κ is due to the vanishing electronic contributions for larger x, as it is discernible in Figures S10 and S12 in the Supplemental Material.

Figure 4(b) displays the figure of merit ZT(x) of Cd_3-*x*_Zn_*x*_As_2_. At 300 K, the enhancement of $S^{2}/\rho$ [Figure 3(c)] and the suppression of κ [Figure 4(a)] lead to two peaks in ZT(x). The first peak is observed at x=0.6, for which ZT is close to 0.2. The second peak appears at x=1.0, where ZT reaches ∼0.15. For x>1.0, ZT is almost negligible because of the large resistivity values measured for these samples around 300 K.

At 600 K, The first peak is again observed at x=0.6 while the second peak has shifted to x=1.2. In both cases ZT exceeds 0.5. The main driving ingredient in the former case is the power factor, while in the latter case the strong suppression of the thermal conductivity at intermediate x provides the biggest impact. Hence, the drop of ZT across x=0.8 at both temperatures is mainly due to the fading of the power factor in this x range while the thermal conductivity has not yet been reduced sufficiently to compensate for it. For x>1.2, ZT is strongly suppressed at 600 K, taking its lowest value at x=2.0. Upon further increasing x, a slight increase to almost ZT∼0.1 appears, which is mainly due to an again enhanced thermopower with x at elevated temperatures in combination with the still fairly small thermal conductivity.

**Figure 5.**
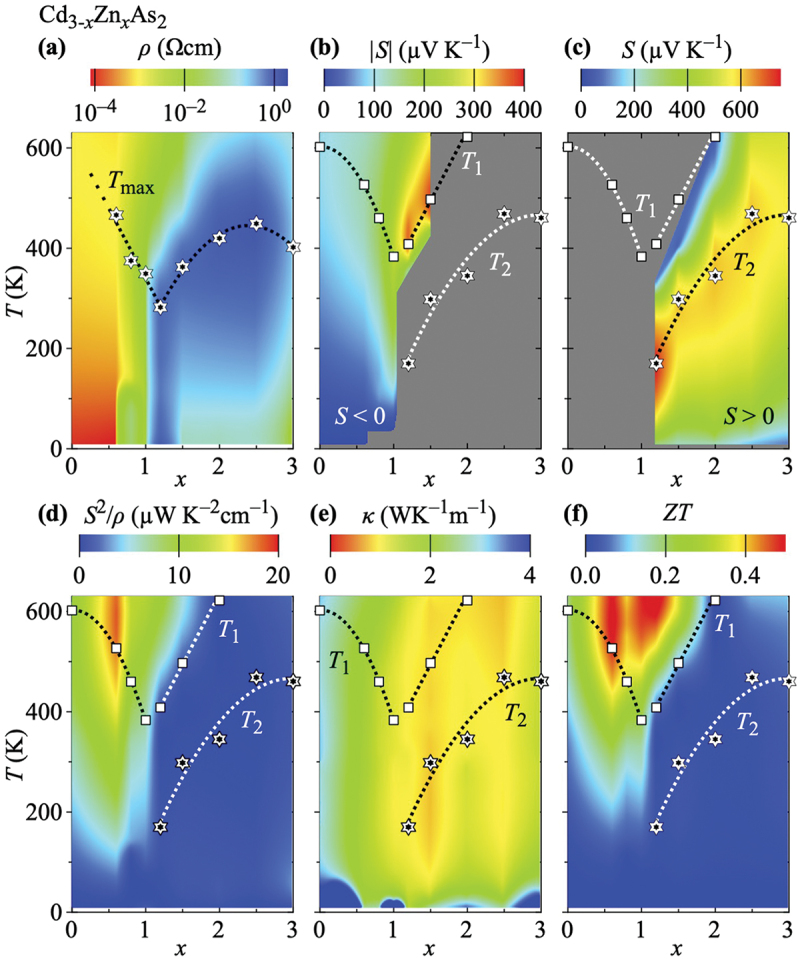
Contour plots of all properties relevant to the figure of merit in Cd_3-*x*_Zn_*x*_As_2_ as a function of the Zn concentration x (horizontal axis) and the temperature T (vertical axis): (a) resistivity ρ, (b) and (c) absolute thermopower |S| for S<0 and S>0, respectively (note the different scale bars), (d) power factor $S^{2}/\rho$, (e) thermal conductivity κ, and (f) figure of merit ZT. In panel (a), the maxima in the temperature dependence of the resistivity ($T_{\max}$) and in panels (b) – (f) the respective extrema in temperature-dependent thermopower data ($T_{1}$ and $T_{2}$) are superimposed. The color code in all panels is chosen so that red and blue indicate advantageous and disadvantageous for enhancing ZT, respectively.

Complementary contour plots are presented in Figure 5, which allow to easily perceive the overall evolution of the thermoelectric quantities and how they contribute to ZT in Cd_3-*x*_Zn_*x*_As_2_. The resistivity is shown in Figure 5(a). Its strong increase up to x∼2.5 at elevated temperatures and in the whole x range at low temperatures are clearly revealed.

Figures 5(b,c) depict the absolute values of the thermopower |S| for S<0 (smaller x) and S>0 (larger x), respectively. The initial increase of |S| with x and T, when starting to introduce Zn, is traceable in Figure 5(b). The thermopower takes its largest absolute values at intermediate x as indicated by the two red hot spots in the central upper half of Figure 5(b) and the central lower half of Figure 5(c), where the shift of $T_{2}$ with x toward higher temperatures accompanied by a slow reduction of |S| is well resolved. The contour plot of the power factor $S^{2}/\rho$ shown in Figure 5(d) peaks around x=0.6 as functions of x and T, making it apparent how ZT(x) is enhanced at elevated temperatures for x<∼1.2.

The plot of the thermal conductivity in Figure 5(e) reveals how κ is suppressed with x and T and that it remains small almost up to x=3.0, where some thermal conductivity is regained. The resulting figure of merit is shown in Figure 5(f), where the second peak in ZT(x) around x=1.2 and at elevated temperatures is revealed and can be easily traced back to the concomitant suppression of κ.

### 3.2. Topological aspects of the band structure

Finally, we discuss whether the anticipated changes in the band structure when alloying the Dirac semimetal Cd_3_As_2_ with the trivial semiconductor Zn_3_As_2_ are reflected in our experimental observations shown in Figures 2–4. To this end, we theoretically calculated the thermopower as a function of temperature and for different charge carrier concentrations on the basis of the band structures for Cd_3_As_2_ and Zn_3_As_2_, presented in Figures 1(b,c), respectively. These assume the β structural phase $P4_{2}/\mathrm{nmc}$ which is realized for 0.6≤x≤1.2. The $\beta^{\prime }$*-phase*, which forms for 1.5≤x≤2.5, has a superstructure in which the length of the crystallographic c axis is doubled, cf. Section S1 in the Supplemental Material. The band structures as well as the band gaps are likely to be similar in both phases and, hence, also the resulting thermopower. Moreover, in the calculations to determine $S_{\mathrm{calc}}$ for Zn_3_As_2_, the band gap was reduced to 50% of its obtained value (0.76 eV) by shifting the conduction bands in order to mimic the band structure for the intermediate region particularly around 1.2≤x≤1.5, where the band gap is expected to be smaller than that of the end compound Zn_3_As_2_. It should also be noted that we apply the constant-τ (scattering time) approximation while the energy dependence of τ may contribute to S significantly near the anticipated change in the band structure.

**Figure 6.**
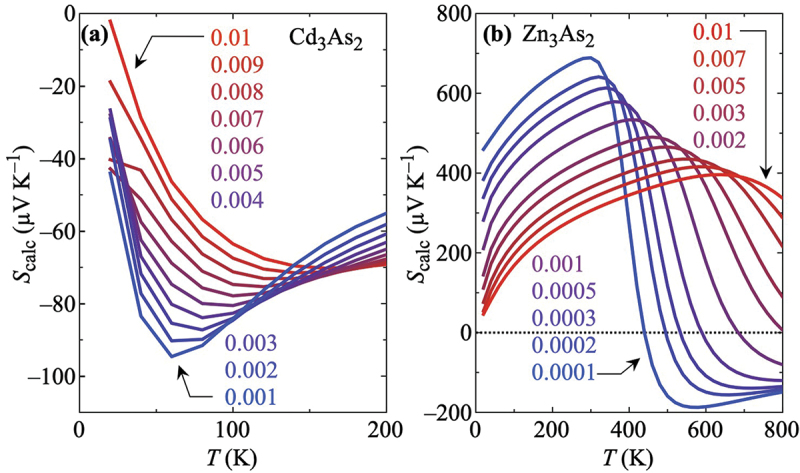
Calculated temperature dependence of the thermopower $S_{\mathrm{calc}}$ of (a) Cd_3_As_2_ and (b) Zn_3_As_2_ for different charge carrier densities [electrons in (a) and holes in (b), respectively] under the assumption of a rigid-band shift of the Fermi level. The change in the carrier count is indicated by the coloring (from large ↔ red to small ↔ blue). This reduction corresponds to the effect of the increase of the Zn (Cd) concentration in the experiment when starting from Cd_3_As_2_ (Zn_3_As_2_). The carrier counts are given in (a) in electrons per unit cell (corresponding to approximately $10^{18}cm^{-3}\;-\;10^{19}cm^{-3}$) and in (b) in holes per unit cell ($10^{17}cm^{-3}\;-\;10^{19}cm^{-3}$; 0.001 per unit cell corresponds to approximately $1\times 10^{18}cm^{-3}$).

Figures 6(a,b) summarize the temperature dependencies of the calculated thermopower $S_{\mathrm{calc}}$ for several charge carrier concentrations for Cd_3_As_2_ and Zn_3_As_2_, respectively. The change in the carrier count in the calculations mimics the observed change in the experimental carrier concentration induced by alloying Cd_3_As_2_ and Zn_3_As_2_, as shown in Figure 3(d): Upon partially replacing Cd with Zn, the electron concentration is reduced while the hole concentration decreases upon introducing Cd into Zn_3_As_2_ toward the intermediate x range.

A comparison of $S_{\mathrm{calc}}$ and the experimental results $S_{\exp}$ shown in Figure 2(b) reveals striking similarities: In the case of Cd_3_As_2_ [Figure 6(a)], the theoretical curve with the largest electron concentration has to be compared with the experimental data for x=0: It is negative and exhibits an extremum as seen in the experimental thermopower in Figure 2(b). Upon reducing the carrier count, the absolute values $|S_{\mathrm{calc}}|$ increase and the temperature of the extrema decreases, as seen in $S_{\exp}$ as a function of x in Figure 2(b). Hence, there are qualitative and quantitative similarities between theory and experiment for 0≤x≤1.0 when the thermopower is calculated for Cd_3_As_2_ with a rigid-band assumption.

As for the opposite end compound Zn_3_As_2_, the theoretical curve with the largest hole concentration in Figure 6(b) has to be compared with the experimental data for x=3.0, both of which exhibit a broad extremum. Upon reducing the carrier count, lower-T extrema develop in $S_{\mathrm{calc}}(T)$ and the temperatures of the higher-T extrema decrease. Remarkably, the theoretical data for the smallest carrier counts almost perfectly reproduce the experimental results for x=1.2 and 1.5 shown in Figure 2(b). Moreover, the calculations for Zn_3_As_2_ do not only produce the order of magnitude of $S_{\exp}$ but also the temperature scale fits well. Apparently, these theoretical data on Zn_3_As_2_ fit qualitatively and quantitatively to the experimental results for 1.2≤x≤3.0.

These observations provide hints that the physics in the solid solution Cd_3-*x*_Zn_*x*_As_2_ for 0≤x≤1.0 is likely governed by a band structure of the same type as realized in the Dirac semimetal Cd_3_As_2_ [Figure 1(b)], while for 1.2≤x≤3.0 it is of the same type as in Zn_3_As_2_ [Figure 1(c)], i.e. the expected changes in the band structure take place between x=1.0 and 1.2. Notably, several other properties also exhibit a significant change in this x range. These are summarized and discussed in Section S6 in the Supplemental Material and therein shown in Figure S17.

This finding also allows to speculate why the mobility μ shown in Fig 3(e) features a maximum at small Zn concentrations. The small effective mass $m^{*}$ for x ∼ 1.0 − 1.2 with the chemical potential lying near the Dirac point should become larger when the chemical potential is raised upon increasing the charge carrier concentration, i.e. decreasing x, cf. Figure 3(d). Hence, the mobility $\mu \propto \tau /m^{*}$ with the scattering time τ should decrease. However, experimentally the opposite is initially observed: μ increases upon decreasing x. This can be understood if the intrinsic disorder due to the mixture of Cd and Zn is taken into account (cf. the discussion in Section S7 in the Supplemental Material): It is reasonable that the reduced disorder enhances τ, overcompensating the expected effect of the effective mass enhancement upon decreasing x. When the disorder gets more and more reduced upon approaching Cd_3_As_2_, at some x this overcompensation will fade out and μ can be expected to exhibit a maximum, which we observe around x ∼ 0.6.

We close the discussion by noting that such a strongly temperature-dependent thermopower, including sign changes and the appearance of one or two extrema was also reported for Bi${\;}_{1-x}$Sb${\;}_{x}$ alloys, cf., e.g., [61,62]. This alloy is also bridging between semiconducting and semimetallic states and hosts the first experimentally confirmed three-dimensional topological insulator as a function of x [63,64], showing a qualitative resemblance with the present Cd_3-*x*_Zn_*x*_As_2_.

## 4. Conclusion

To gain control over the thermoelectric figure of merit, this work follows the strategy to start with a topologically nontrivial system, here the Dirac semimetal Cd_3_As_2_, and to alloy it with a trivial band insulator, namely its lighter counterpart Zn_3_As_2_. Both materials possess very different physical properties (e.g., metal vs. semiconductor, electron- vs. hole-type conduction, high vs. low mobility). Therefore, studying such a solid solution is a promising approach to gain fine-tuned control over the resistivity ρ, the thermopower S, the thermal conductivity κ, and, hence, the figure of merit $\mathrm{ZT}=S^{2}T/(\rho \kappa )$. The present work demonstrates that this approach has great potential to be successful: At elevated temperatures, the figure of merit of Cd_3-*x*_Zn_*x*_As_2_ exhibits two different x ranges around x=0.6 and 1.2, where ZT exceeds ∼ 0.5. While the first maximum in ZT(x) can be traced back to an enhancement of the power factor, the second maximum is mainly caused by the suppression of the thermal conductivity.

A comparison of experimental and theoretical thermopower data calculated as a function of temperature for various charge carrier concentrations points toward a scenario that the topological band structure present in the Dirac semimetal Cd_3_As_2_ changes to trivial between x=1.0 and 1.2. This is further supported by the observation that several other properties in this solid solution also exhibit significant changes in this x range. This work underlines that topologically nontrivial systems are not only fascinating because of their intriguing physics, but that it can also be very promising and valuable to probe their thermoelectric performance, especially when bridging toward trivial systems by alloying other elements.

## 5. Methods

### 5.1. Sample growth and characterization

Polycrystalline batches of Cd_3-*x*_Zn_*x*_As_2_ with x=0, 0.6, 0.8, 1.0, 1.2, 1.5, 2.0, 2.5, and 3.0 were grown by conventional melt growth. For each batch, inside a glove box stoichiometric amounts of the respective elements (shots of Cd: purity 6N, Asahi Chemical Co. LTD., Japan; Zn: 5N, Nilaco Corp., Japan; As: 7N5, Furukawa Co. LTD., Japan) were loaded into carbonized quartz tubes. These were provisionally closed and transferred to a pumping station where they were evacuated and eventually sealed. Then they were composition-dependently fired to be melted for 48 h to 72 h at 950${\;}^{\circ }$C to 1050${\;}^{\circ }$C and eventually slowly cooled back to room temperature. The phase purity of all batches was checked with an in-house powder x-ray diffractometer (XRD, Rigaku, Japan). Our results are in qualitative agreement with the structural phase diagram reported in Ref. [52]. A reproduction of this phase diagram is shown in Figure S1 in the Supplemental Material. The concentrations x examined in this work are highlighted therein. The results of the XRD measurements are shown in Figures S2 and S3 along with the lattice constants as a function of x in Figure S4. Table S1 provides an overview of the different structural phases existing in this solid solution and their relationship.

The chemical composition of all batches was checked with a scanning-electron microscope equipped with an energy-dispersive x-ray analyzer (SEM-EDX, JEOL, Japan, and Bruker, USA). Resulting images for selected x are shown in Figures S5 – S7, and the results of the EDX analyses are summarized in Figure S8 in the Supplemental Material. Throughout the text the nominal Zn concentration x is used when referring to samples.

### 5.2. Measurements

Various measurements at low and at elevated temperatures are necessary to determine the figure of merit. Some of them require different sample geometries. Therefore, for each x, several samples were cut from the respective as-grown batches.

### 5.2.1 Low-temperature measurements

The measurements of the longitudinal ρ and the Hall resistivity $\rho_{\mathrm{yx}}$ were performed by a conventional five-probe method in a commercial cryostat (physical property measurement system PPMS, Quantum Design, USA), employing the standard resistivity option. The analysis of the Hall resistivity is described in detail in Section S4 in the Supplemental Material. The thermopower S and the thermal conductivity κ were measured in homebuilt setups fitting into a PPMS. A temperature gradient was applied to the long side of the respective sample and the voltage difference /temperature gradient was measured by commercial thermocouples, cf. Ref. [58].

### 5.2.2 High-temperature measurements

Resistivity and thermopower were measured by employing a commercial apparatus (ZEM-3, Advance Riko, Japan) with the samples kept in He atmosphere and held by two Ni electrodes acting simultaneously as current leads. One of them is equipped with a heater to apply a temperature gradient. Two thermocouples acted as voltage pads in the resistivity measurements and were used to monitor the temperature gradient during the subsequent thermopower measurements. They were mechanically pushed onto a polished sample surface. The same samples used for the high-temperature ρ and S measurements were also used for the respective measurements at low temperatures, which were performed after the high-temperature experience.

The thermal conductivity above room temperature was calculated via $\kappa =\lambda \;c_{p}\;d$ with the thermal diffusivity λ, the specific heat $c_{p}$, and the sample density at room temperature d as estimated from the XRD data taken on each batch. To ensure that the XRD density is reliable, we roughly crosschecked it by also estimating the density of each sample from its mass and volume (from the linear dimensions). These two densities agree within ±3%. The thermal diffusivity was measured in N${\;}_{2}$ atmosphere by employing the laser-flash method in a commercial system (LFA-457, Netzsch, Germany). For this purpose, the polished top and bottom surfaces of the samples were coated with graphite spray. The specific heat was measured in Ar atmosphere in a commercial system by means of differential scanning calorimetry (STA449 F1 Jupiter, Netzsch, Germany).

The misfits between the low- and high-temperature data around room temperature are within 9.5% (resistivity), 8.5% (thermopower), and 15% (thermal conductivity).

We note that the upper limit of the temperature range studied here is due to the decomposition or degradation of Cd_3-*x*_Zn_*x*_As_2_ at elevated temperatures, as also reported earlier [65,66]. The exact temperature range where the system dissociates differs between different studies. Our own analyses on powder samples suggest that a detectable mass loss sets in above 650 K.

Comments concerning the known issues about the reproducibility of transport data in Cd_3_As_2_ (see, e.g., Refs. [34,67]) and the comparison of the present work with our preceding study [58] can be found in Section S7 in the Supplemental Material. We note for clarity that all batches were newly grown and that no data published in our previous study [58] are used in the present work while the examined temperature and Zn concentration ranges are largely extended.

### 5.3. Computational details

Electronic-structure calculations were performed in the space group $P4_{2}/\mathrm{nmc}$ with the reported lattice constants for Cd_3_As_2_ [68] and Zn_3_As_2_ [69] by using the Vienna Ab initio Simulation Package (VASP) [70]. For Cd_3_As_2_, the projector-augmented wave (PAW) method [71,72] was employed with the exchange correlation functionals of the Perdew-Burke-Ernzerhof (PBE) type [73] in agreement with a previous calculation [32] and for Zn_3_As_2_ the modified Becke-Johnson (mBJ) type [74,75], respectively. By using mBJ, we obtain a band gap of 0.76 eV for Zn_3_As_2_, which is basically consistent with previous calculations [76,77]. Spin-orbit coupling is included in all the calculations using VASP. We utilized the BoltzWann module [78] of the Wannier90 package [79] to calculate the thermopower using a k-point mesh of 100×100×100 and employ the constant-τ approximation, i.e. the energy dependence of the scattering rate is neglected for simplicity.

## Supplementary Material

Supplemental Material
