# Supplementary material for: Enhancement of the thermoelectric figure of merit in the Dirac semimetal Cd3As2 by band-structure and -filling control
Source: Sci Technol Adv Mater. 2024 Oct 8;25(1):2412971. doi: 10.1080/14686996.2024.2412971 (PMC11639228; doi:10.1080/14686996.2024.2412971)
Supplement: Supplemental Material [file TSTA_A_2412971_SM6817.pdf]

Supplemental Material for "Enhancement of the Thermoelectric Figure of Merit in the Dirac Semimetal Cd_3As_2 by Band-Structure and -Filling Control"

Markus Kriener,[1,*] Takashi Koretsune,[2] Ryotaro Arita,[1,3]
Yoshinori Tokura,[1,4,5] and Yasujiro Taguchi[1]

[1] *RIKEN Center for Emergent Matter Science (CEMS), Wako 351-0198, Japan*
[2] *Department of Physics, Tohoku University, Miyagi 980-8578, Japan*
[3] *Research Center for Advanced Science and Technology, University of Tokyo, Tokyo 153-8904, Japan*
[4] *Department of Applied Physics and Quantum-Phase Electronics Center (QPEC), University of Tokyo, Tokyo 113-8656, Japan*
[5] *Tokyo College, University of Tokyo, Tokyo 113-8656, Japan*

(Dated: September 25, 2024)

This Supplemental Material provides additional data on $Cd_{3-x}Zn_xAs_2$ as follows:

- Section S1: About the crystal structures in $Cd_{3-x}Zn_xAs_2$
- Section S2: Results of SEM-EDX analyses
- Section S3: Thermal conductivity data
- Section S4: Analysis of the Hall resistivity for $x = 1.2$ and 1.5
- Section S5: Analysis of the longitudinal resistivity at high temperatures
- Section S6: Comparison of additional properties
- Section S7: Sample dependence of transport data in $Cd_{3-x}Zn_xAs_2$

* corresponding author: markus.kriener@riken.jp

S1. About the crystal structures in $Cd_{3-x}Zn_xAs_2$

Structural phase diagram from Ref. [1]

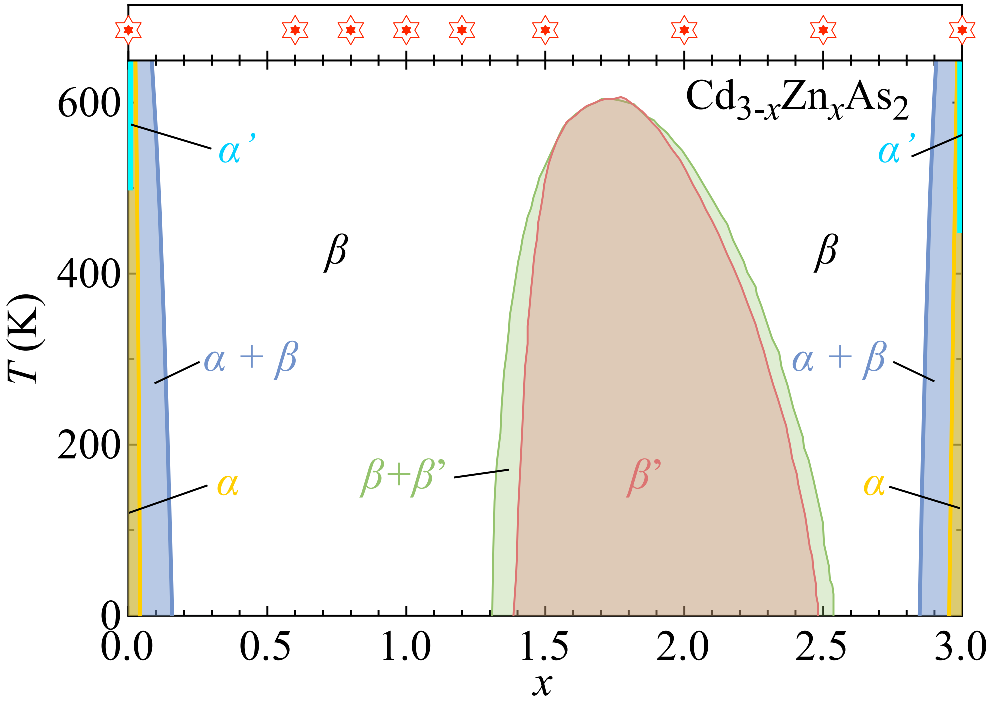

FIG. S1. Structural phase diagram replotted from Ref. [1]. For the definition of the different phases see text. The red star symbols at the top indicate the nine different Zn concentrations studied in this work.

Figure S1 is replotted from Ref. [1] and provides an overview of the complex situation on the crystal structures realized in $Cd_{3-x}Zn_xAs_2$. In addition, the Zn concentrations of the samples studied in this work are indicated by red star symbols at the top of the diagram.

$Cd_{3-x}Zn_xAs_2$ exhibits structural phase transitions as a function of x and temperature. The different structures relevant here are all tetragonal and summarized in Table S1. Therein, the tetragonal lattice constants a and c are given in relation to the structural modification labeled α, and Z denotes the number of formula units per unit cell. Note that the labels for these structural modifications are not used consistently in the literature. The present notation follows Ref. [1]. In addition to these listed in Table S1, for Cd_3As_2 there are also two additional high-temperature modifications ($T > 650$ K) mentioned in the literature: α'' (tetragonal) and a cubic one. For Zn_3As_2, only the cubic phase is reported [1, 2, 5].

TABLE S1. **Different crystal structures realized in $Cd_{3-x}Zn_xAs_2$.**

Label	Crystal Structure	Space Group	Lattice Parameter		Z	Reference
α	$I4_1/acd$	142	a	c	32	[1, 2]
	$I4_1/cd$	110	a	c	32	[3, 4]
α'	$P4_2/nbc$	133	$\approx a$	$\approx c$	32	[5]
β	$P4_2/nmc$	137	$a/\sqrt{2}$	$c/2$	8	[1, 6]
β'	$I4_1/amd$	141	$a/\sqrt{2}$	c	16	[1, 7]

According to our own measurements, the system starts to dissociate above approximately 650 K, for which reason these are omitted here. The dissociation process is also mentioned in [5, 8].

X-ray diffraction patterns

In this subsection the x-ray diffraction (XRD) patterns taken at room temperature of all nine studied batches are discussed. Since both pristine compounds Cd_3As_2 and Zn_3As_2 take a different structure as compared to the alloyed crystals, the XRD patterns are split into Figures S2 and S3. Figure S2 presents XRD data of Cd_3As_2 in panel (c) and Zn_3As_2 in (d) normalized to the respective main peaks. The expected peak positions for the two settings discussed in the literature are shown for Cd_3As_2 in (a) $I4_1/cd$ and (b) $I4_1/acd$ and for Zn_3As_2 in (e) and (f), respectively.

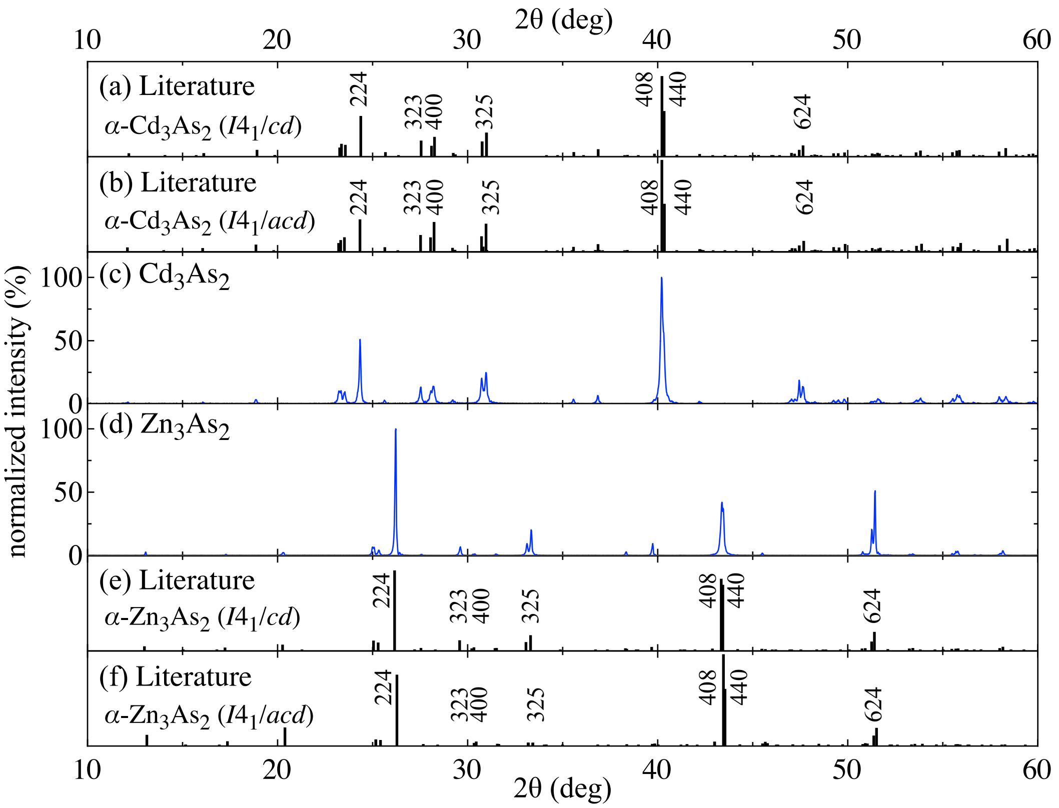

FIG. S2. Normalized XRD data of the two pristine compounds (c) Cd_3As_2 and (d) Zn_3As_2. The expected peak positions for both structures discussed in the literature are shown for Cd_3As_2 in (a) $I4_1/cd$ and (b) $I4_1/acd$ and those for Zn_3As_2 in (e) and (f), respectively. Stronger reflections are labeled.

Figure S3 summarizes XRD data for the seven alloyed batches of $Cd_{3-x}Zn_xAs_2$ ($0.6 \leq x \leq 2.5$) normalized to the respective main peaks. Panels (a) and (i) show the expected peak positions for Cd_3As_2 and Zn_3As_2 in the β phase $P4_2/nmc$, (j) provides the expected peak positions for the β' structure $I4_1/amd$ for a sample with $x = 2.2$ taken from a recent publication [7] which can be readily compared with the data in panel (g). According to Ref. [1], the β' structure is realized in samples with $\sim 1.35 \leq x \leq \sim 2.5$. In our own data, tiny indications are already traceable in the XRD data for $x = 1.2$, see e.g., the shoulder formation at the 202_β peak which is split in the case of $x = 1.5$. In the data for $x = 2.5$ this feature is still clearly discernible but already somewhat weaker. This probably indicates the reappearance of the β phase at higher x. In panel (g) the positions of selected peaks belonging to the β' structural modification are indicated by red diamond symbols. Based on the data at hand, it is difficult to judge whether the samples with $1.5 \leq x \leq 2.5$ completely crystallize in the β' phase because the two strongest peaks of each structural modification (202_β, 224_β and $204_{\beta'}$, $400_{\beta'}$) appear at similar 2θ angles.

In summary, we qualitatively confirmed the published structural phase diagram shown in Fig. S1. The structural transitions from α to α' observed in the end compounds Cd_3As_2 and Zn_3As_2 as a function of temperature are also confirmed quantitatively. These produce clear anomalies in specific-heat data (not shown) and are within 3% (Cd_3As_2) and 8% (Zn_3As_2) of the published temperature values. The phase bounderies of the β' dome seem slightly different according to our XRD data but this does not affect the conclusions drawn in this work.

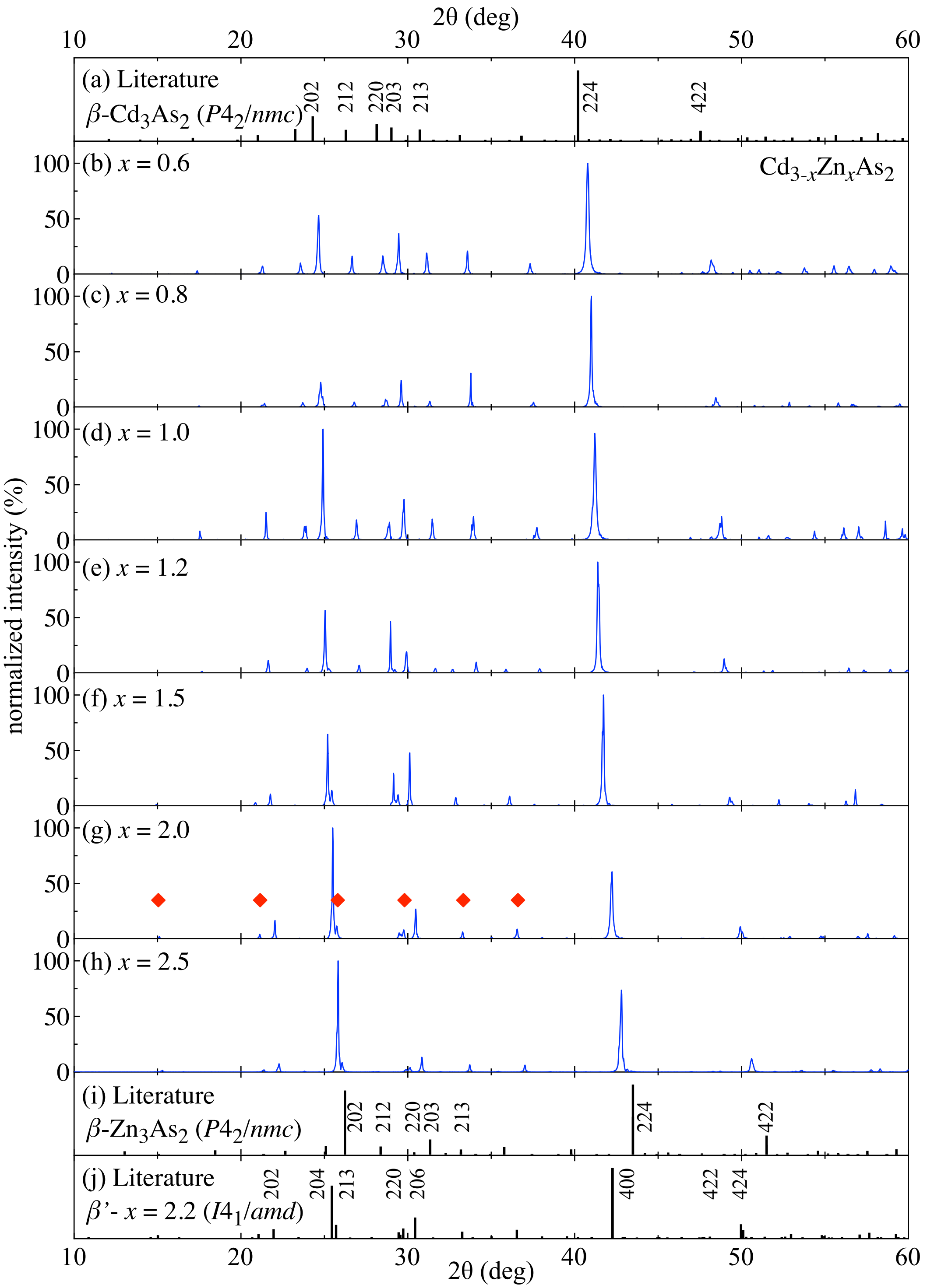

FIG. S3. Normalized XRD data of the seven alloyed compounds $Cd_{3-x}Zn_xAs_2$ with $0.6 \leq x \leq 2.5$ studied here. The expected peak positions for β-Cd_3As_2 and β-Zn_3As_2 are shown in (a) and (i), respectively. Panel (j) shows the peak positions of a sample with $x = 2.2$ in the β' structure from Ref. [7] for comparison. In panel (g), some of the peaks belonging to β' are indicated with red diamonds. Stronger reflections are labeled. See text for details.

Lattice parameters

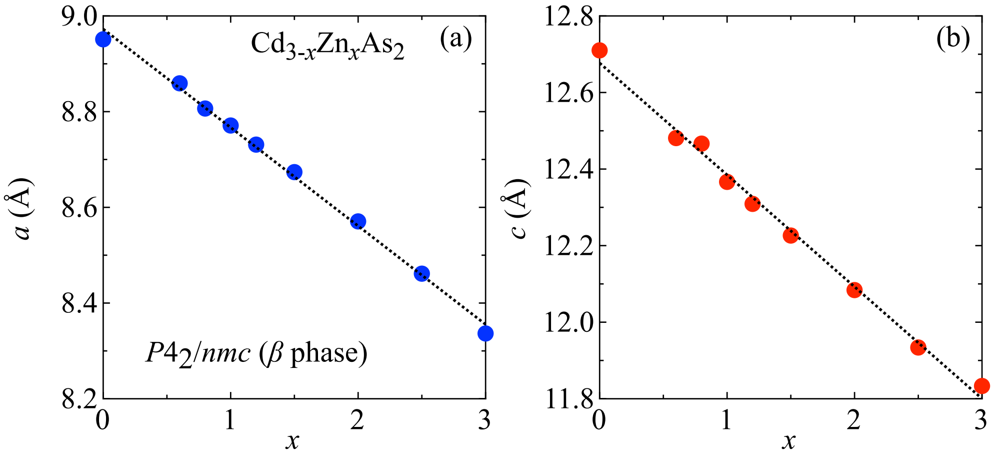

FIG. S4. Lattice parameters of the (a) a and (b) c axis given in the setting of the β structural modification.

Figure S4 provides the x dependence of the tetragonal lattice constants a and c of $Cd_{3-x}Zn_xAs_2$ in the setting of the β phase. Both decrease approximately linearly with x, satisfying Vegard's law.

S2. Results of SEM-EDX analyses

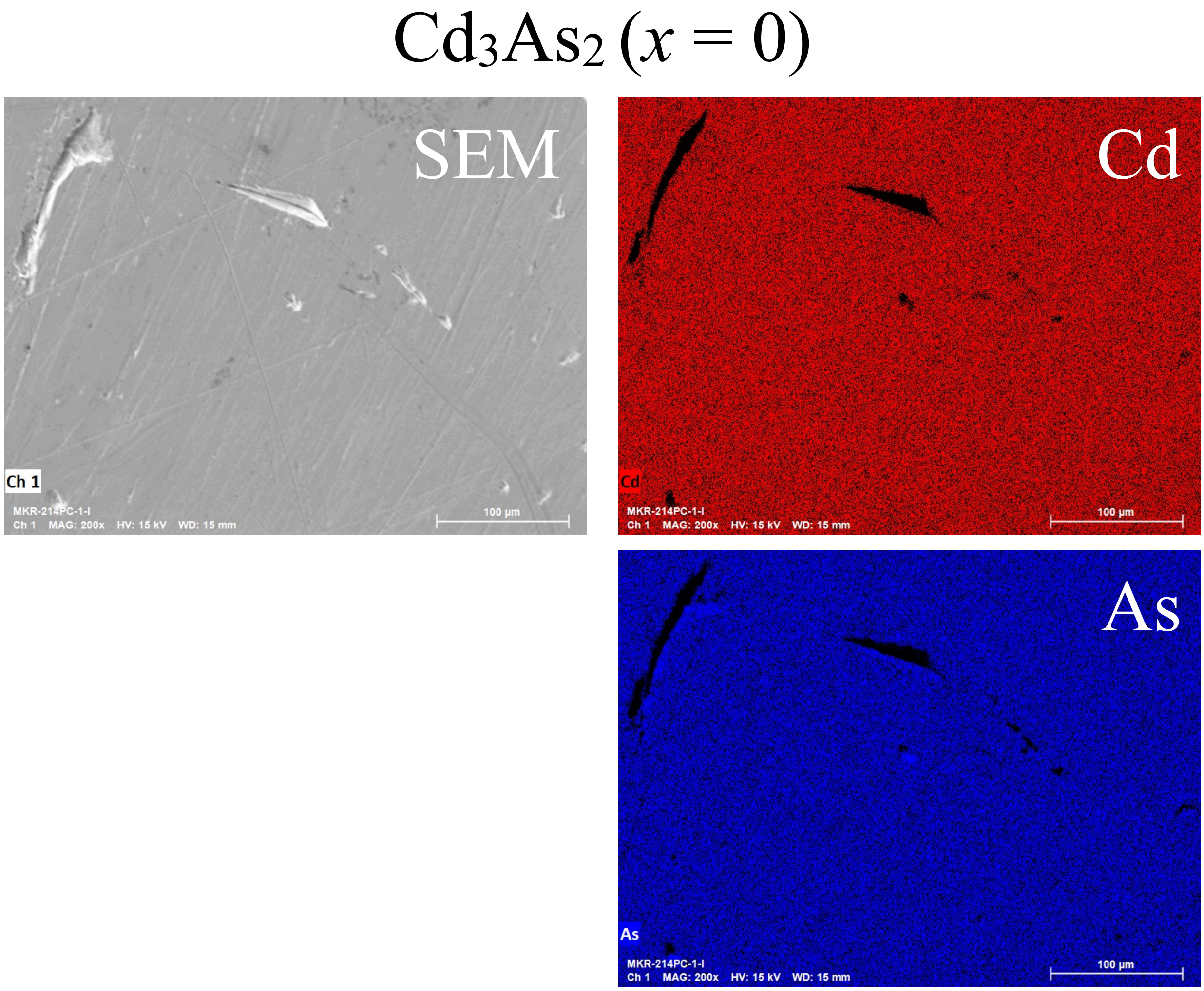

FIG. S5. SEM-EDX images for Cd_3As_2 ($x = 0$). The scale bar in all images indicates 100 μm.

All batches studied were analyzed by SEM-EDX. Selected resulting images are shown in Figs. S5 ($x = 0$), S6 ($x = 1.5$), and S7 ($x = 3.0$). These indicate homogeneous distributions of all elements. EDX analyses were carried out on up to 30 spots in different surface areas of a sample. The chemical composition of each spot was estimated assuming that the As concentration is 2. The averaged results are shown in Figure S8 for (a) the Cd concentration, (b) the Zn concentration, and (c) the sum of both. The experimental Cd (Zn) concentration decreases (increases) roughly linearly with the nominal Zn concentration. The sum of the Cd and Zn concentrations slightly exceeds 3 in both pristine materials and falls slightly below in case of the alloyed samples. This deviation from the nominal value is getting smaller with

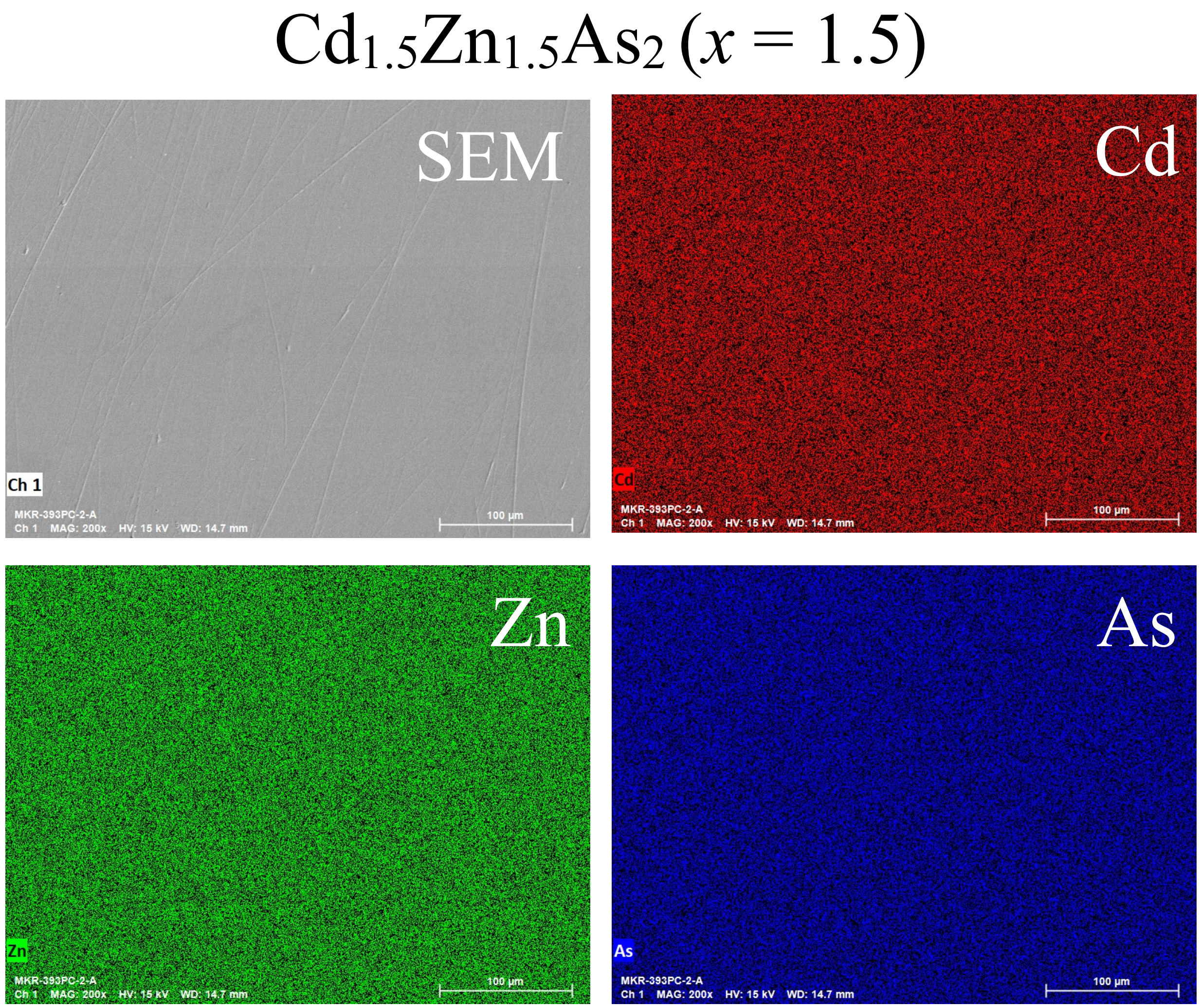

FIG. S6. SEM-EDX images for $Cd_{1.5}Zn_{1.5}As_2$ ($x = 1.5$). The scale bar in all images indicates 100 μm.

x. The error bars are given by the standard deviation calculated from the averaged results of the various EDX analyses and are small throughout the solid solution.

Zn_3As_2 (x = 3.0)

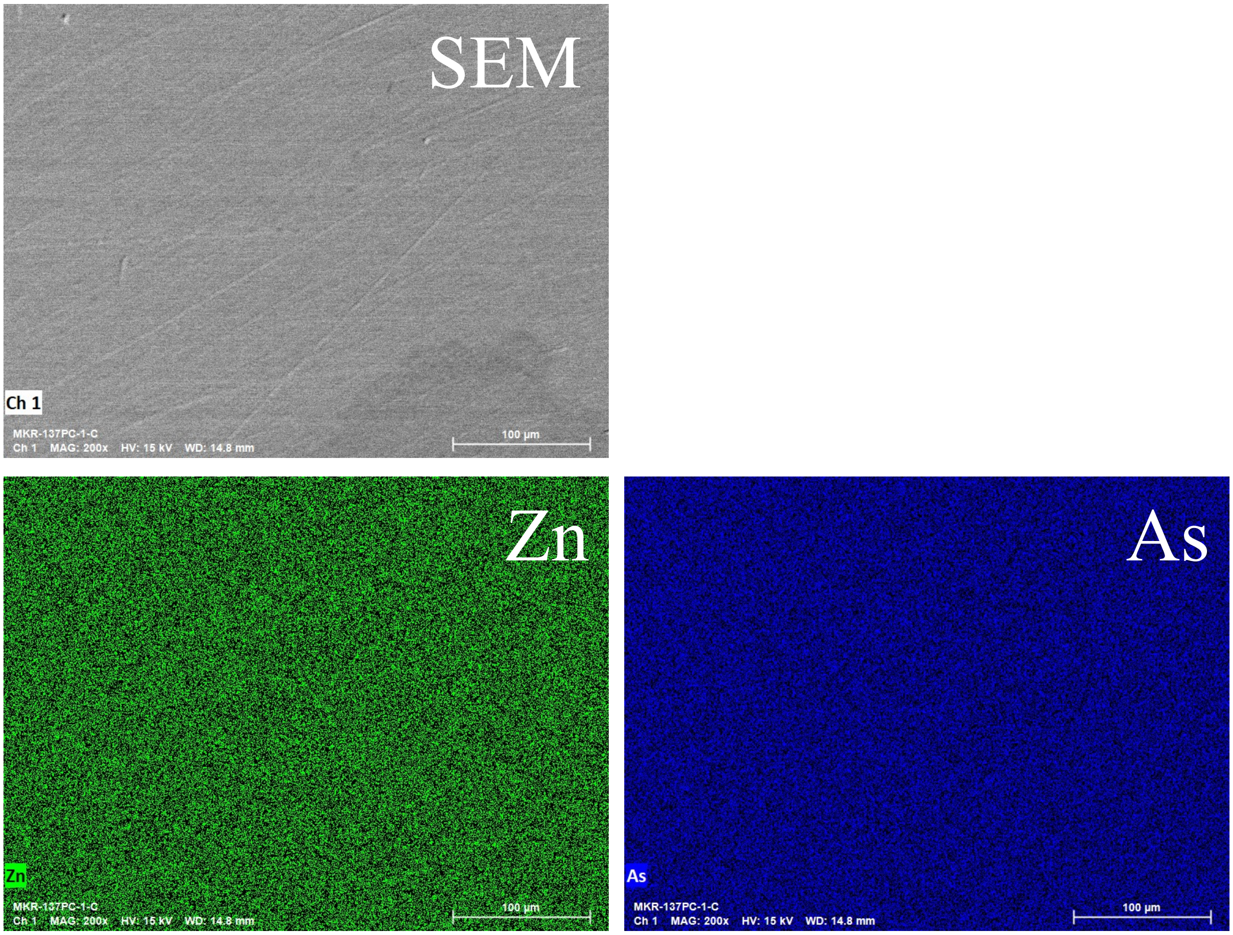

FIG. S7. SEM-EDX images for Zn_3As_2 ($x = 3.0$). The scale bar in all images indicates 100 μm.

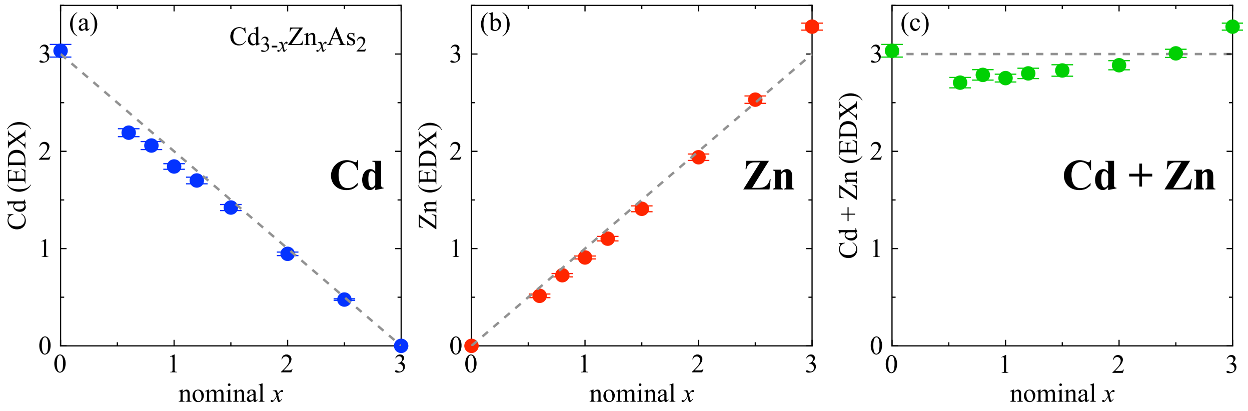

FIG. S8. Results of EDX analyses of $Cd_{3-x}Zn_xAs_2$: Estimated chemical compositions of (a) Cd, (b) Zn, and (c) Cd + Zn assuming that the As concentration is 2. The dashed lines in all panels indicate the respective stoichiometric concentration.

S3. Details of the thermal conductivity

In this Section, the thermal conductivity data for all studied samples are shown in Fig. S9, including the data which are omitted in Figure 2(d) of the main text. As discussed therein, the overall thermal conductivity κ is very small and further systematically suppressed as a function of x with a slightly recovered κ for $x = 3.0$. However, the Umklapp peak at low temperatures is suppressed for the alloyed samples as compared to the pristine compounds except for $x = 1.0$. Conceivable reasons are inhomogeneous samples or differences in the crystal lattice. As argued in Section S2, we can rule out the former. As for the latter, a structural transition below room temperature is unlikely [9]. However, the ideal chemical formula of this system is Cd_4As_2, hence, 25% of the Cd sites are voids [2]. Upon alloying Cd_3As_2 with Zn_3As_2 Cd, Zn, and the voids have to be arranged on the Cd sites, increasing the disorder. This could cause an additional suppression of the Umklapp peak as compared to the two pristine compounds. For samples in between with an integer amount of Cd and Zn, such as $x = 1$, some additional ordering ("superstructure") of Cd, Zn, and the voids may be possible, leading to a (partial) recovery of the Umklapp peak. We note that also for $x = 2.0$, a larger Umklapp peak is observed although not as pronounced as for $x = 0$, 1.0, or 3.0.

The total thermal conductivity $\kappa = \kappa_{el} + \kappa_{ph}$ summarized in Fig. S9 consists of contribu-

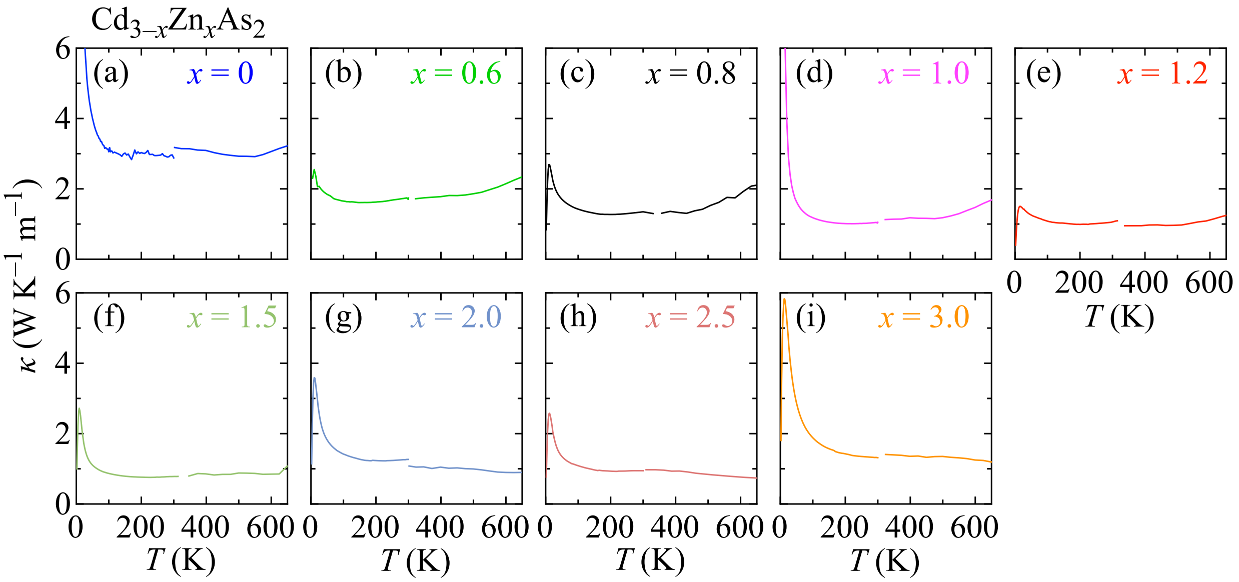

FIG. S9. Total thermal conductivity in $Cd_{3-x}Zn_xAs_2$.

tions from the charge carriers κ_{el} and the lattice κ_{ph}. Assuming that the Wiedemann-Franz law $\kappa_{\mathrm{el}} = L_0 T/\rho$ with the Lorenz number $L_0 = 2.44 \times 10^{-8}$ V^{2}K^{-2} holds in this system, one can separate them. The resulting breakdown is shown in Figures S10 (κ_{el}) and S11 (κ_{ph}). Data for $x = 1.2$ and 1.5 are excluded in the latter because in these samples both carrier types, electron and holes, are obviously present and contribute to the electronic and heat transport. In this case, the simple Wiedemann-Franz law does not allow to quantitatively separate these due to contributions from the bipolar thermal conductivity (see, e.g., [10]). Respective plots of the x dependencies of κ_{el} and κ_{ph} are shown in Fig. S12.

Apparently, the electronic contributions are strongly suppressed with x in agreement with the disappearance of the high-mobility electrons [Figures 3(d) and 3(e) of the main text]. The low-mobility holes which start to appear across $x = 1.2$ do not contribute much to κ. Along with the overall small phononic contributions, the total thermal conductivity is small, which is the main source of the second maximum in $ZT(x)$, as discussed in the main text.

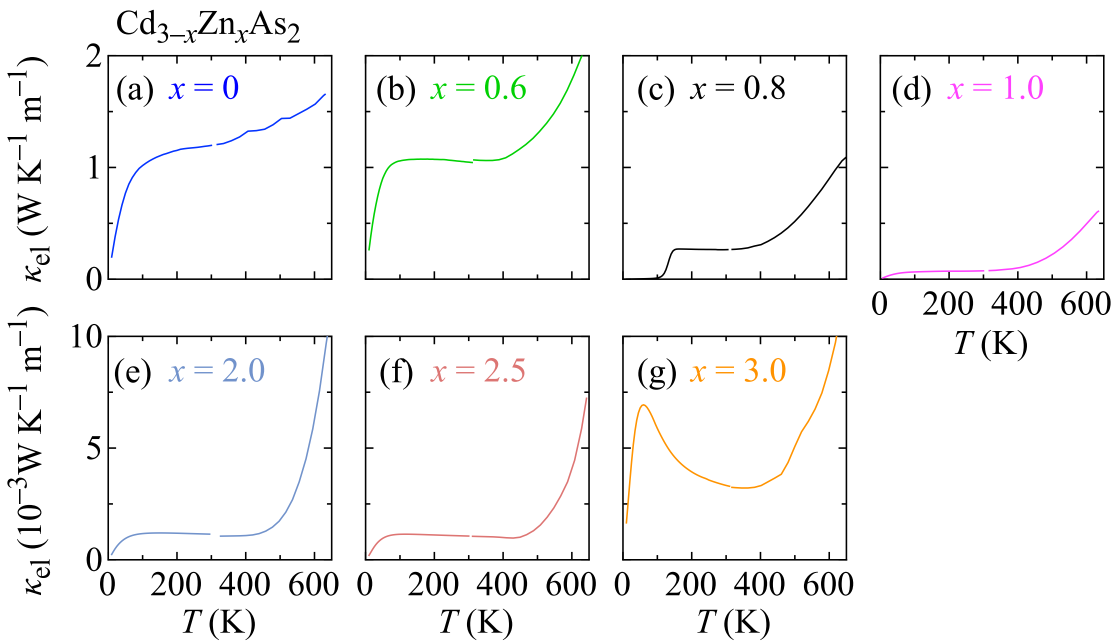

FIG. S10. Contribution of the charge carriers κ_{el} to the total thermal conductivity in $Cd_{3-x}Zn_xAs_2$. Note the differences in the ordinate scale of panels (a) – (d) and (e) – (g).

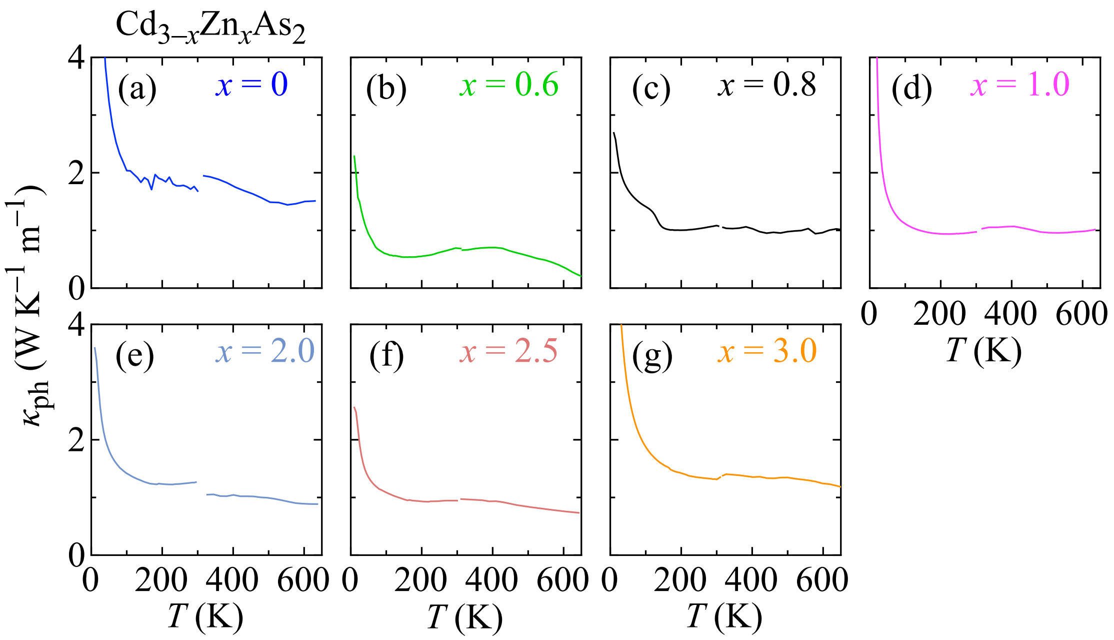

FIG. S11. Contribution of the phonons κ_{ph} to the total thermal conductivity in $\mathrm{Cd}_{3-x}\mathrm{Zn}_x\mathrm{As}_2$.

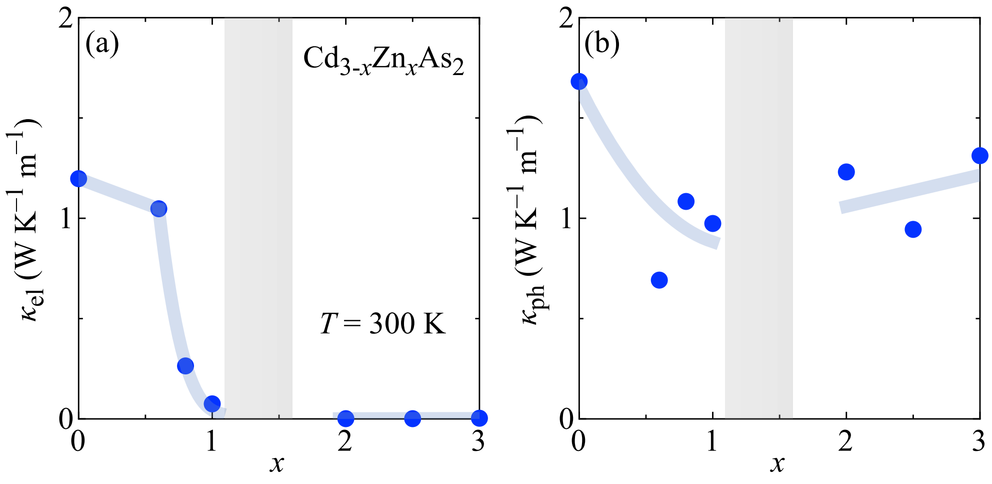

FIG. S12. Zn concentration dependence of (a) the electronic and (b) the phononic contributions to the total thermal conductivity $\kappa = \kappa_{\mathrm{el}} + \kappa_{\mathrm{ph}}$ of $\mathrm{Cd}_{3-x}\mathrm{Zn}_x\mathrm{As}_2$ $(0 \le x \le 3)$ at $T = 300$ K. These were obtained by assuming that the Wiedemann-Franz law $\kappa_{\mathrm{el}} = L_0 T/\rho$ with the Lorenz number $L_0 = 2.44 \times 10^{-8}$ $\mathrm{V}^2\mathrm{K}^{-2}$ holds in this solid solution. The grey shaded areas indicate the x range where this law cannot be used due to the simultaneous appearance of electron and hole charge carriers. Respective data points for $x = 1.2$ and 1.5 are therefore omitted, see text. In both panels, blueish bold lines are guides to the eyes.

S4. Analysis of the Hall resistivity for $x = 1.2$ and 1.5

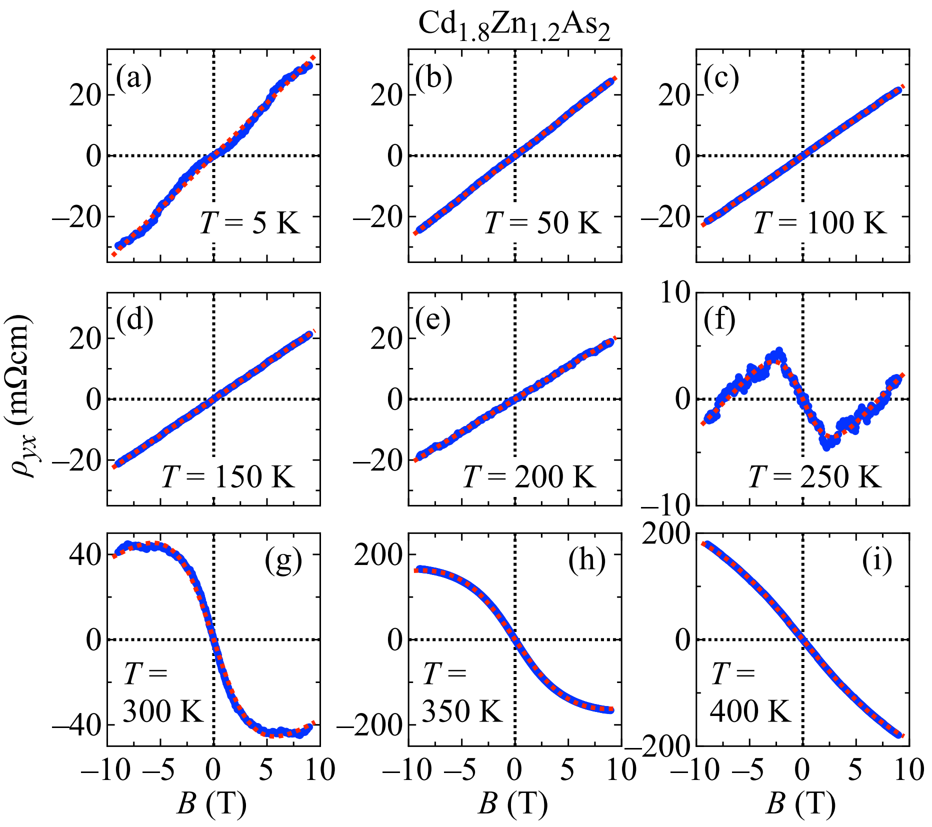

FIG. S13. Magnetic field B-dependent Hall resistivity ρ_{yx} of $Cd_{1.8}Zn_{1.2}As_2$ at selected temperatures between 5 K and 400 K. Red dotted lines are fits to the data, see text.

In this subsection, the Hall resistivity and its fitting of $Cd_{1.8}Zn_{1.2}As_2$ (Figure S13) and $Cd_{1.5}Zn_{1.5}As_2$ (Figure S14) are discussed.

For all samples, the Hall resistivity $\rho_{yx}(B)$ (B denotes the magnetic field) was measured at selected temperatures between 5 K and 400 K and antisymmetrized with respect to B prior to the analysis. Except for the samples with $x = 1.2$ and 1.5, $\rho_{yx}(B)$ is either linear with a negative ($x \leq 1.0$) or with a positive slope ($x \geq 2.0$), implying that the charge transport is mainly dominated by one carrier type. The respective charge carrier concentrations were estimated from a simple linear (one-channel) fit. For the two samples with $x = 1.2$ and 1.5, the main carrier type changes from electrons to holes as a function of x and temperature. As shown in Figures S13 and S14, at low temperatures, the conduction in both samples is hole-type: $T \leq \sim 200$ K for $x = 1.2$ and $T \leq \sim 250$ K for $x = 1.5$, respectively. Above, the Hall resistivity is not linear but curved, indicative of two-channel charge transport from

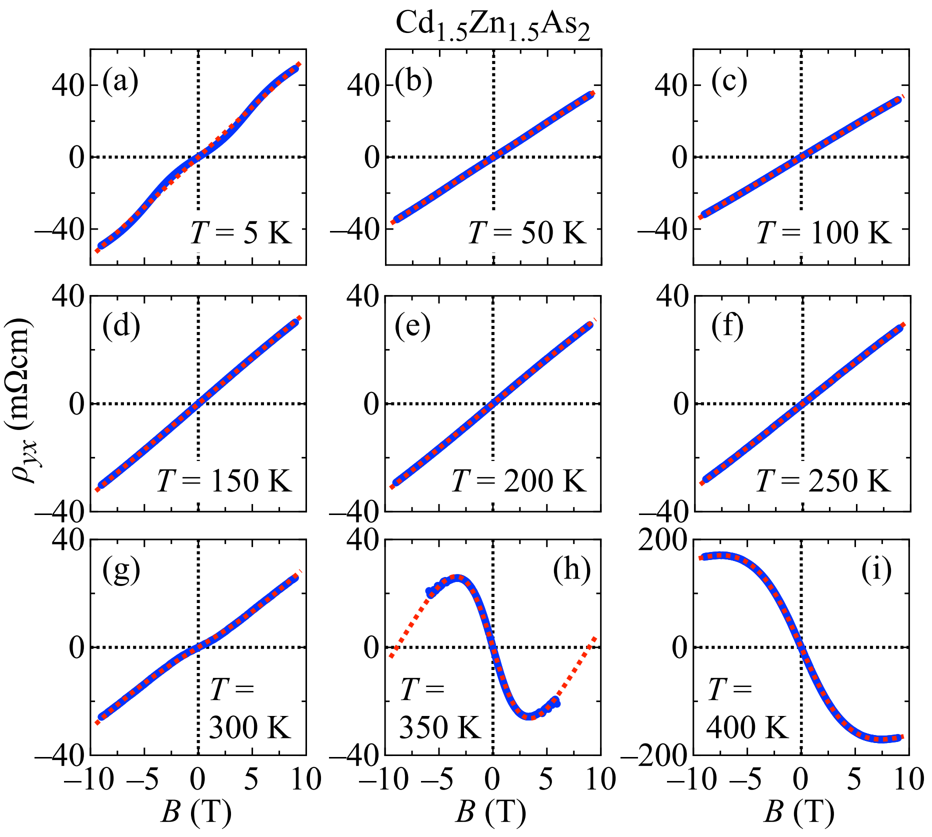

FIG. S14. Magnetic field B-dependent Hall resistivity ρ_{yx} of $Cd_{1.5}Zn_{1.5}As_2$ at selected temperatures between 5 K and 400 K. Red dotted lines are fits to the data, see text.

electrons and holes. To fit these $\rho_{yx}(B)$ data, the two-channel model given in Equation 1 was employed [11]:

$$\rho_{yx}(B) = R_{\mathrm{H}} \cdot B = \frac{\mu_{\mathrm{e}}^2 n_{\mathrm{e}} + \mu_{\mathrm{h}}^2 n_{\mathrm{h}} + (\mu_{\mathrm{e}}\mu_{\mathrm{h}}B)^2(n_{\mathrm{e}} + n_{\mathrm{h}})}{((\mu_{\mathrm{e}}|n_{\mathrm{e}}| + \mu_{\mathrm{h}}n_{\mathrm{h}})^2 + (\mu_{\mathrm{e}}\mu_{\mathrm{h}}B)^2(n_{\mathrm{e}} + n_{\mathrm{h}})^2)\, e} \cdot B. \tag{1}$$

Here, R_{H} denotes the Hall constant, n_{e}, μ_{e} and n_{h}, μ_{h} the charge carrier concentrations n_{i} and mobilities μ_{i} of the electrons (index e) and holes (index h), and $e = 1.6 \times 10^{-19}$ C the elementary charge. In this notation $n_{\mathrm{e}} < 0$ and $n_{\mathrm{h}} > 0$. Moreover, one of the four fitting parameters (μ_{e}) was fixed by the simultaneously measured longitudinal resistivity $\rho_{xx}(B = 0)$ via

$$\rho_{xx}(0) = \frac{1}{(|n_{\mathrm{e}}|\mu_{\mathrm{e}} + n_{\mathrm{h}}\mu_{\mathrm{h}})\, e}. \tag{2}$$

This approach yielded the red dotted fitting curves in Figures S13 and S14.

The temperature dependence of the respective charge carrier concentrations and mobilities for both samples are summarized in Figure S15: The n_{i} are shown in the top panels

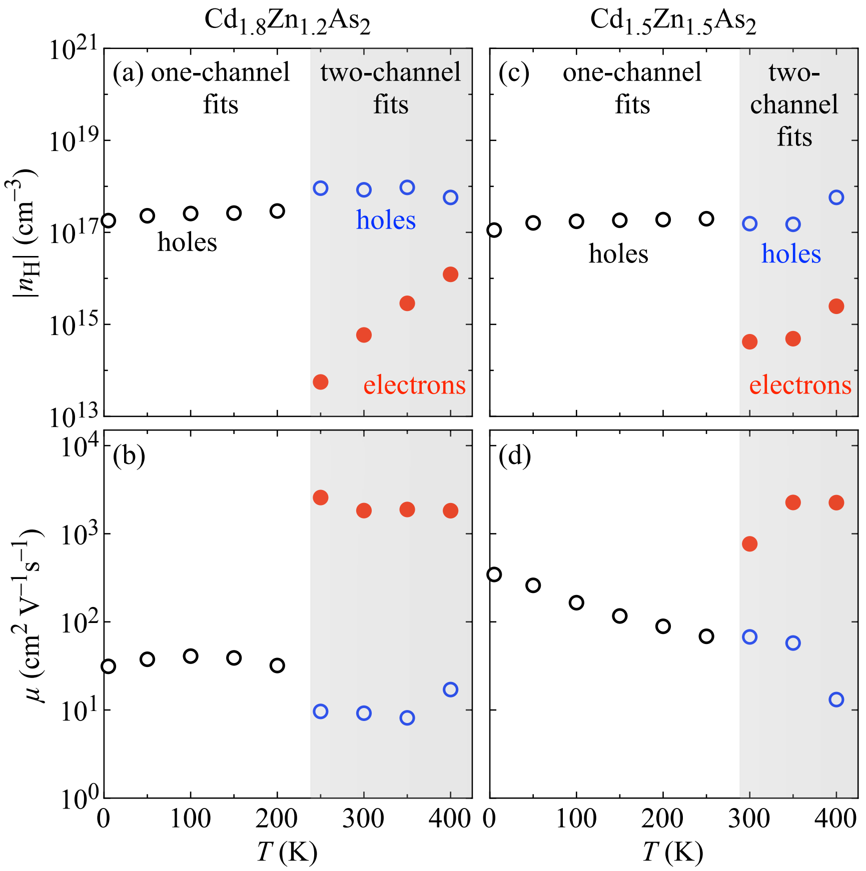

FIG. S15. Temperature dependence of the (a), (c) charge carrier concentrations and (b), (d) mobilities as estimated from fitting Equation 1 to the Hall resistivity $\rho_{yx}(B)$ of (a) and (b) $Cd_{1.8}Zn_{1.2}As_2$ and (c) and (d) $Cd_{1.5}Zn_{1.5}As_2$. Open symbols refer to hole-type, filled symbols to electron-type conduction. One-channel fit results are given in black, two-channel results in blue (holes) and red (electrons). The gray shaded area in each panel indicates the temperature range in which both carrier types obviously coexist.

(a) $x = 1.2$ and (c) $x = 1.5$, the μ_i in the bottom panels (b) $x = 1.2$ and (d) $x = 1.5$, respectively. In both samples the electron concentration increases with temperature but on a very low level $< \sim 10^{16}$ cm^{-3}, i.e., almost depleted while they keep their high mobility $> \sim 10^3$ cm^{2}V^{-1}s^{-2}. Holes dominate the charge transport with a concentration of $10^{17} - 10^{18}$ cm^{-3} but exhibit a much smaller mobility $< \sim 10^2$ cm^{2}V^{-1}s^{-2}.

S5. Analysis of the longitudinal resistivity at high temperatures

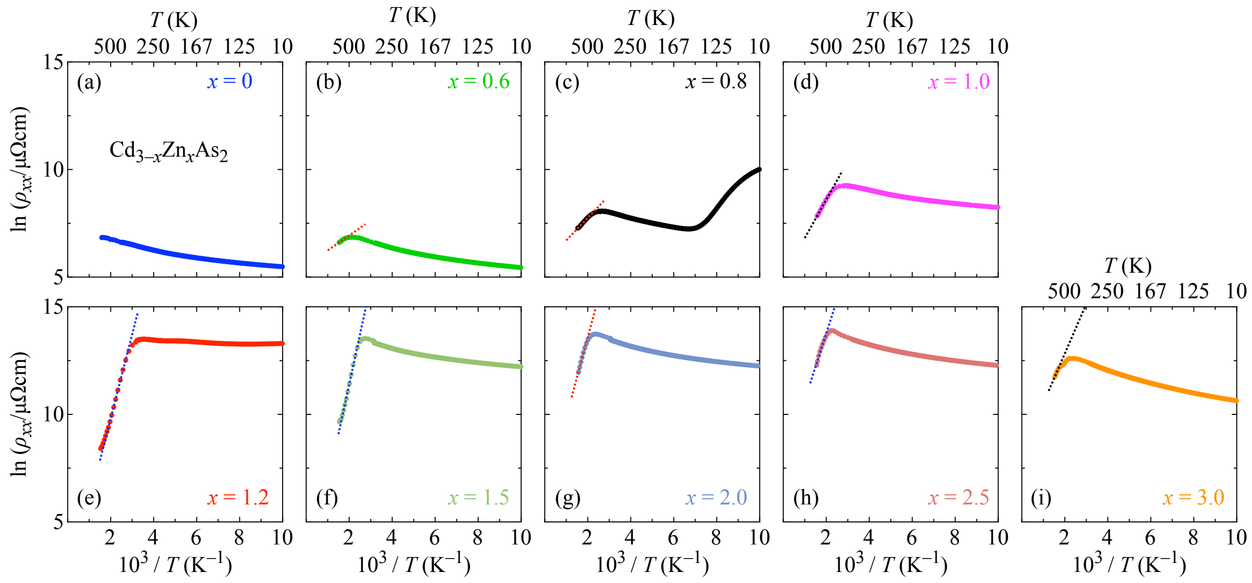

FIG. S16. Longitudinal resistivity of $Cd_{3-x}Zn_xAs_2$ displayed as Arrhenius plots $\ln \rho_{xx}$ vs T^{-1}. The top axes give the temperature in K. The dotted lines in panels (b) – (i) are linear fits to the high-temperature part of the resistivity.

Figure S16 displays the longitudinal resistivity as Arrhenius plots $\ln \rho_{xx}$ vs T^{-1} along with linear fits (dotted lines in the panels for $x \geq 0.6$) to the high-temperature part of ρ_{xx} above the maximum in the data shown in Figure 2(a) of the main text. From the slopes of the linear fits, the respective activation energies $\Delta_{\mathrm{Arr}}(x)$ were estimated. These are discussed along with a comparison with other characteristic properties in the next Section.

S6. Comparison of additional properties

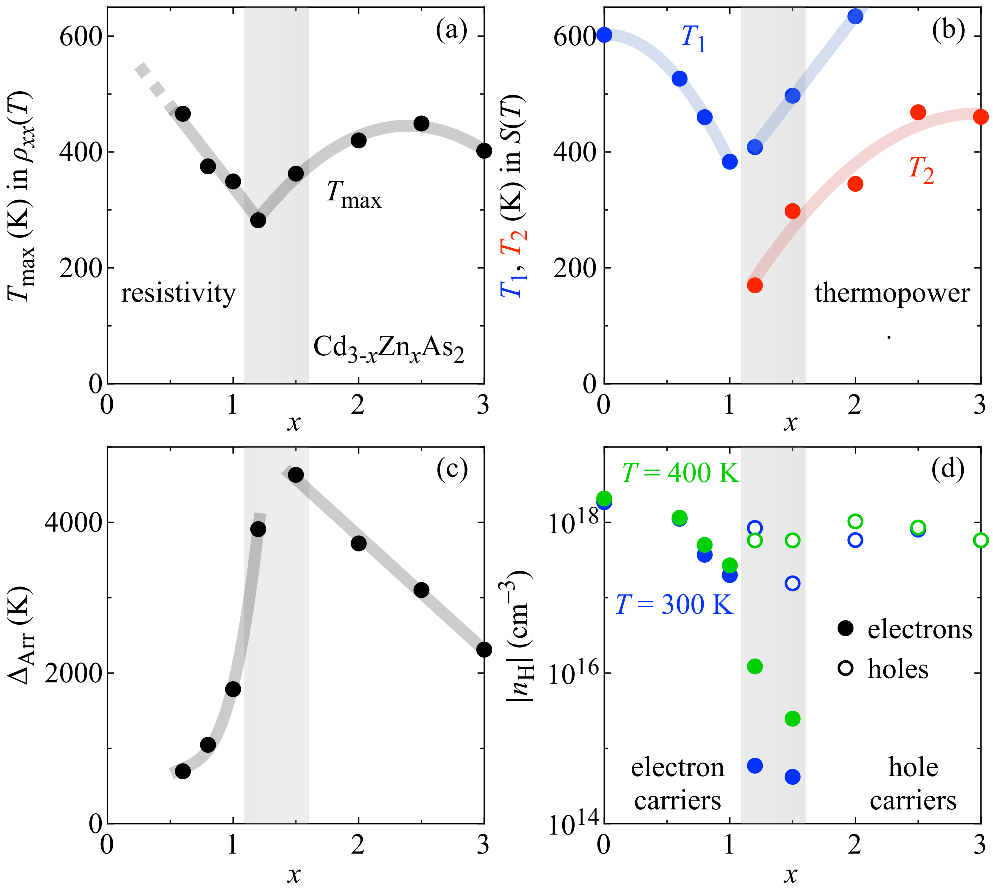

FIG. S17. Zn concentration dependence of (a) the temperature T_{max} of the maxima in resistivity data, (b) the temperatures T_1 (blue) and T_2 (red) of the extrema in thermopower data, (c) the activation energy Δ_{Arr} as estimated from the linear fits shown in Figure S16, and (d) the absolute value of the charge carrier concentration $|n_H|$ estimated from Hall-resistivity data at 300 K (blue) and 400 K (green). In the latter, filled and open symbols indicate electron- and hole-type conduction, respectively. The gray shaded area in each panel indicates the crossover range $\sim 1.2 \leq x \leq \sim 1.5$ where the dominant carrier type changes from electrons to holes as discernible in (d). The bold lines in panels (a) – (c) are guide to the eyes.

Figure S17 summarizes the x dependence of several characteristic features observed in $Cd_{3-x}Zn_xAs_2$. Panel (a) shows the x dependence of the extrema T_{max} in the temperature dependence of the resistivity [Figure 2(a) of the main text], which decrease linearly with x up to $x \sim 1.2$. Above, $T_{max}(x)$ starts to increase and peaks around $x \sim 2.5$.

Figure S17(b) shows the respective extrema T_1 and T_2 observed in the temperature dependence of the thermopower data [Figure 2(b) of the main text]: The extrema T_1 decrease up to $x = 1.0$ and increase above. For $x \geq 1.2$, $T_2(x)$ in $S(T)$ evolves qualitatively similar as T_{max} in $\rho(T)$. However, the extrema T_2 are missing in $S(T)$ for $x \leq 1.0$.

The x dependence of the activation energy Δ_{Arr} as estimated from the Arrhenius plots shown in Figure S16 is plotted in Figure S17(c). It exhibits an increase with x for $x \leq 1.2$. By contrast, for $x \geq 1.5$ it decreases linearly with x.

Figure S17(d) summarizes the x dependence of the absolute charge carrier concentration $|n_{H}|$ as estimated from Hall-resistivity measurements at 300 K and 400 K, with the latter being the highest temperature at which we can measure $\rho_{yx}(B)$. The 300-K data are replotted from Figure 3(d) of the main text. Comparing these data, for most samples the charge carrier concentration slightly increases as a function of temperature.

As for the situation below $x \sim 1.2$, the existence of maxima in $\rho(T)$ indicates that there are additional thermally activated charge carriers at elevated temperatures. These are reflected in the increase in n_{H} at a given x when going from 300 K to 400 K. It is probably safe to assume that this tendency holds up to higher temperatures, given the overall behavior of the resistivity shown in Figures 2(a) and 3(a) in the main text. Together with the decrease in $|n_{H}(x)|$, this suggests that the Fermi level shifts downwards with x up to at least $x \sim 1.0$. As seen in the band structure plot in Figure 1(a) of the main text, there are indeed additional bands within a few 100 meV of E_F, to which carriers can be excited in qualitative agreement with this picture. However, this scenario cannot explain the behavior observed upon further increasing the Zn concentration: the x dependencies of the properties shown in Figures S17(a)–(d) change drastically, pointing toward a significant modification of the band structure.

Altogether, these findings further support the conclusion drawn in the main text that the band structure up to $x \sim 1.0$ is of the Cd_3As_2 type, i.e., topologically nontrivial, and that this changes across $x \sim 1.2$, beyond which the system is dominated by the topologically trivial band structure of the Zn_3As_2 type. We note that even in nominally pure Zn_3As_2, the

Fermi level does not lie inside the band gap but in the valence bands due to unintentionally doped holes. Upon increasing x above 1.2, E_F shifts slightly downwards toward the end compound Zn_3As_2 as indicated by the increase in the hole concentration.

S7. Sample dependence of transport data in $Cd_{3-x}Zn_xAs_2$

As for the comparison with our previous publication [9], the present results are qualitatively in good agreement. However, quantitatively this is not always the case in resistivity and thermopower measurements as, e.g., manifested in the occasional observation of a metal-insulator transition below room temperature in samples with $x \leq 1.2$, cf. Ref. [9]. The origin seems twofold:

(i) We observe a strong sample dependence even among samples cut from the same batch, which had been also noticed in the literature, see, e.g., Refs. [12–14]. A possible explanation can be given when taking into account that there is an intrinsically large number of defects in Cd_3As_2 (and $Cd_{3-x}Zn_xAs_2$). As argued in Section S3, chemically the stoichiometric formula of this system should be Cd_4As_2 and, hence, 25% of the Cd sites are empty [2, 15]. It was proposed that the ordering of these voids in Cd_3As_2 can differ from sample to sample [2, 13, 16] causing the differences. From our own experience with this system, we can add here that the results of resistivity and thermopower measurements can vary quantitatively even when measuring on the same sample surface, e.g., when the electric contacts were made on different positions on the same surface. This effect seems to become even worse when introducing Zn on the Cd site.

(ii) We also observe an annealing effect above room temperature, which tends to affect the resistivity and the thermopower and modify their temperature dependencies. This explains the slightly smaller room-temperature values found here for ZT as compared to our previous work, which was restricted to $T < 300$ K.

---

[1] W. Żdanowicz, K. Lukaszewicz, and W. Trzebiatowski, Crystal Structure of the Semiconducting System $Cd_3As_2-Zn_3As_2$, Bull. Acad. Pol. Sci. **12**, 169 (1964).

[2] M. N. Ali, Q. Gibson, S. Jeon, B. B. Zhou, A. Yazdani, and R. J. Cava, The Crystal and Electronic Structures of Cd_3As_2, the Three-Dimensional Electronic Analogue of Graphene, Inorg. Chem. **53**, 4062 (2014).

[3] G. A. Steigmann and J. Goodyear, The Crystal Structure of Cd_3As_2, Acta. Cryst. B **24**, 1062 (1968).

[4] A. Pietraszko and K. Lukaszewicz, The Crystal Structure of Zinc Arsenide Polymorphic Mod-

ifications α-Zn_3As_2 and α'-Zn_3As_2., Bull. Acad. Pol. Sci. **24**, 459 (1976).

[5] A. Pietraszko and K. Lukaszewicz, Thermal Expansion and Phase Transitions of Cd_3As_2 and Zn_3As_2., Phys. Status Solidi A **18**, 723 (1973).

[6] W. Żdanowicz, F. Królicki, and P. Plenkiewicz, Preparation and semiconducting properties of pseudobinary solid solutions Zn_3As_2-Zn_3P_2, Acta Phys. Pol. A **44**, 447 (1973).

[7] G. Volodina, V. S. Zakhvalinskii, and V. K. Kravtsov, Crystal structure of $\alpha'''-(Zn_{1-x}Cd_x)_3As_2$ ($x = 0.26$), Cryst. Reports **58**, 563 (2013).

[8] J. B. Westmore, K. H. Mann, and A. W. Tickner, Mass Spectrometric Study of the Nonstoichiometric Vaporization of Cadmium Arsenide, J. Phys. Chem. **68**, 606 (1964).

[9] J. Fujioka, M. Kriener, D. Hashizume, Y. Yamasaki, Y. Taguchi, and Y. Tokura, Alloying-induced enhancement of thermopower in the Dirac-semimetal system $Cd_{3-x}Zn_xAs_2$, Phys. Rev. Mater. **5**, 094201 (2021).

[10] A. F. May, E. S. Toberer, A. Saramat, and G. J. Snyder, Characterization and analysis of thermoelectric transport in n-type $Ba_8Ga_{16-x}Ge_{30+x}$, Phys. Rev. B **80**, 125205 (2009).

[11] D. Maryenko, A. S. Mishchenko, M. S. Bahramy, A. Ernst, J. Falson, Y. Kozuka, A. Tsukazaki, N. Nagaosa, and M. Kawasaki, Observation of anomalous Hall effect in a non-magnetic two-dimensional electron system, Nat. Commun. **8**, 14777 (2017).

[12] T. Ito, M. Wada, M. Iwami, and K. Kawabe, Two-Band and Impurity-Band Conduction in the $Cd_{3-x}Zn_xAs_2$ Alloy Crystals, J. Phys. Soc. Jpn. **43**, 1672 (1977).

[13] T. Liang, Q. Gibson, M. N. Ali, M. Liu, R. J. Cava, and N. P. Ong, Ultrahigh mobility and giant magnetoresistance in the Dirac semimetal Cd_3As_2, Nat. Mater. **14**, 280 (2014).

[14] I. Crassee, E. Martino, C. C. Homes, O. Caha, J. Novák, P. Tückmantel, M. Hakl, A. Nateprov, E. Arushanov, Q. D. Gibson, R. J. Cava, S. M. Koohpayeh, K. E. Arpino, T. M. McQueen, M. Orlita, and A. Akrap, Nonuniform carrier density in Cd_3As_2 evidenced by optical spectroscopy, Phys. Rev. Lett. **97**, 125204 (2018).

[15] Z. Wang, H. Weng, Q. Wu, X. Dai, and Z. Fang, Three-dimensional Dirac semimetal and quantum transport in Cd_3As_2, Phys. Rev. B **88**, 125427 (2013).

[16] T. Liang (private communication).
